# Identification of rice genotypes for reproductive stage heat tolerance and yield stability through multi-trait and multivariate analysis

**DOI:** 10.3389/fpls.2026.1853773

**Published:** 2026-06-17

**Authors:** Agalya Jasmin, Karthika Rajendran

**Affiliations:** Department of Genetics and Plant Breeding, VIT School of Agricultural Innovation and Advanced Learning, Vellore Institute of Technology, Vellore, Tamil Nadu, India

**Keywords:** heat stress, heat tolerance index, MGIDI, rice, yield stability

## Abstract

**Context:**

High temperature during the flowering phase is a major constraint to stable rice productivity. Heat tolerance cannot be explained solely by yield, as yield is affected by several interacting traits. Therefore, an integrated multi-trait evaluation is essential to identify rice genotypes with superior performance under heat stress.

**Objective:**

The research was designed to identify high-yielding, heat-tolerant rice genotypes and the key traits contributing to yield maintenance under heat stress.

**Methods:**

A total of 80 rice accessions, comprising 45 landraces and 35 improved cultivars, were evaluated across four field environments using an alpha-lattice design across two cropping seasons under normal and heat-stress conditions during 2024–2025. Twenty morphological, physiological, and phenological traits were recorded. Pooled analysis of variance, correlation analysis, path coefficient analysis, principal component analysis, cluster analysis, and Multi-Trait Genotype-Ideotype Distance Index (MGIDI) analysis were performed, while a trait-wise heat tolerance index was applied to examine genotype performance under heat stress conditions.

**Results:**

Pooled ANOVA revealed evident impacts of genotype, environment, and genotype × environment interaction for all traits, indicating substantial genetic variability and contrasting responses to heat stress. High temperature during the reproductive stage adversely affected reproductive and yield-associated traits, with the largest reductions noticed in single plant yield, panicle weight, number of filled grains per panicle, spikelet fertility, and productive tillers. Trait-wise, HTI showed considerable diversity among genotypes, with TKM 9, TRY 1, PR 128, TPS 5, and TRY 5 recording superior performance in single-plant yield. Correlation and path coefficient analyses highlighted harvest index, panicle weight, productive tillers, number of grains per panicle, and filled grains per panicle as major contributors for yield maintenance under stress, whereas delayed flowering and high leaf temperature were unfavorable. Principal component analysis revealed that reproductive efficiency and grain formation explained more variation in heat tolerance than vegetative vigor, while cluster analysis identified Cluster III as the most promising group with superior genotypes under heat stress. MGIDI-based ranking further recognized TRY 1, RNR 15048, TPS 5, Indhurani, and Anna R 4 as promising multi-trait genotypes.

**Conclusions:**

Heat tolerance in rice is governed by multiple interacting traits rather than yield alone. Integrating trait-based analysis with trait-wise HTI and MGIDI improves the identification of superior genotypes under field conditions. The identified genotypes and key traits offer valuable resources for breeding heat-tolerant rice under increasing temperature stress.

## 1 Introduction

Increasing thermal instability and climate variability are reshaping global agricultural systems, shifting the focus from maximizing yield under optimal conditions to sustaining productivity under environmental uncertainty. It was reported that nations with high farming-based populations and climate-sensitive agriculture systems would be at higher risk from rising temperatures and extreme weather (INCCA, 2010). The Green Revolution fundamentally transformed agricultural practices because it produced major increases in food grain production and improved national food security systems (Abrol and Sangar, 2006). However, much of this progress was achieved under relatively stable climatic conditions and intensive external input use. Rising temperatures and frequent extreme weather events now challenge the sustainability and reliability of crop production (Alotaibi, 2023; Datta and Behera, 2022).

Rice (*Oryza sativa L*.) is recognized as the vital nutritional source, serving as a staple food for approximately half to two-thirds of the global population and supporting food and nutritional security. The global area under rice cultivation is approximately 169.88 million hectares, with a production of about 713.10 million metric tons and an average rice yield of 4740 kg ha^−1^ during 2024–2025 (Patel et al., 2025). India generates nearly 150 million metric tons of rice each year, which accounts for 25% of international rice production (USDA, 2025). Climate-related stresses impact agricultural systems, which provide direct and indirect support to large portions of the population. Any decrease in crop yield results in extensive social and economic impacts across society. Therefore, future rice production strategies need to develop crop resilience together with yield improvements because this will enable crops to survive unpredictable agricultural conditions. Rice growth and reproductive development are influenced by temperature, making the crop especially vulnerable when environmental conditions exceed the optimal range. Liao et al. (2011) stated that rice achieves its best growth at temperatures between 25°C and 35°C. Exposure to temperatures above the optimal threshold interferes with several key physiological functions, including pollen viability, spikelet fertility, assimilate translocation, and grain-filling duration (Yadav et al., 2022). High temperature stress during heading and flowering is generally defined as a daily mean temperature exceeding 30°C or a daily maximum temperature exceeding 35°C (Ma et al., 2025). Grain yield may decrease by 7%–8% when temperatures rise (Liu et al., 2023), and a 1°C increase has been linked to a 5%–15% increase in spikelet sterility in major rice-growing regions (Senguttuvel et al., 2022). These yield losses are significant. The productivity of rice could be negatively impacted by a 3°C increase, which could raise sterility above 30% (El-Malky, 2024).

India grows rice during three main seasons, which include kharif, rabi, and summer. The summer season experiences its highest vulnerability because of both water shortages and extreme heat conditions (Satturu et al., 2023). The late rabi to summer months (February–June) in Tamil Nadu, especially in the Vellore region, experience very high daily temperatures between 29°C and 37°C and warm nights of 24°C–26°C (WeatherSprak, 2024). Despite this climatic risk, rice is still cultivated during the summer season in many irrigated areas to utilize available water resources, sustain cropping intensity, and obtain an additional harvest. However, this increases the likelihood of exposing the reproductive stage to damaging high temperatures, making heat tolerance an important target in rice breeding. Grain quality and yield are mainly influenced by the heading-to-grain-filling stage, which lasts 30–40 days (Ma et al., 2025). During this time, heat stress disrupts the translocation of carbohydrates, decreases the individual grain weight and grain number, and slowly reduces the harvest index. These high-temperature episodes often coincide with the reproductive phase, exacerbating spikelet sterility and reducing yield stability (Patel et al., 2025). As a result, cropping cycles are gradually changing due to the increasing frequency of extreme heat episodes, and current cultivars are facing new selection pressures.

Selection methods that only consider yield performance in favorable conditions might miss genotypes with built-in physiological heat-buffering systems. According to Fischer and Wood (1979), the grain yield is a complex and polygenic trait that exhibits strong environmental influence. Therefore, genotypes may not be evaluated for intrinsic physiological tolerance mechanisms if they are solely evaluated for absolute yield reduction. Indices of stress tolerance have been widely adopted to classify genotypes that combine resilience and productivity in challenging environments (Fernandez, 1992; Rosielle and Hamblin, 1981). Among these metrics, the heat tolerance index (HTI) concurrently accounts for yield potential and stability, offering a combined assessment of genotype responses under stressed and non-stressed conditions (Bouslama et al., 1984; Farshadfar et al., 2013).

Heat tolerance in rice cannot be adequately explained by yield performance alone because high temperature influences phenological, morphological, physiological, reproductive, and yield-associated traits simultaneously (Liu et al., 2023; Ma et al., 2025; Hossain et al., 2024). Accordingly, an assessment using multiple contributing traits is required to understand the biological mechanisms of differential genotype responses under stress. From this perspective, the integration of 20 traits in the present study facilitated a broader assessment of heat tolerance by encompassing changes in flowering pattern, plant growth, physiological response, reproductive efficiency, and yield performance. Since heat tolerance is a complex and polygenic trait, multivariate statistical methods are crucial for determining how component traits relate to one another and measuring genetic variation between genotypes. Correlation and path coefficient analyses can be employed to determine the direct and indirect contributions of component traits to yield under stress conditions (Dewey and Lu, 1959). While Mahalanobis D^2^ statistics make it easier to cluster genetically heterogeneous genotypes for parental selection, Principal Component Analysis (PCA) lowers dimensionality and identifies key drivers of phenotypic variance (Jolliffe, 2002). The Multi-trait Genotype-Ideotype Distance Index (MGIDI) serves as an effective approach for selecting superior genotypes based on multiple traits concurrently (Olivoto and Nardino, 2021). By estimating each genotype’s distance from an ideal trait value-based ideotype, MGIDI, in contrast to standard selection indices, enables balanced enhancement of yield, stability, and physiological traits. In breeding programs aimed at complex stress responses, this approach improves efficiency and reduces selection bias toward a particular trait.

Although reproductive stage heat tolerance has been widely studied in rice, many earlier screening approaches have mainly been based on yield reduction or only a few traits, which may not completely reflect the complexity of heat stress response. High temperature affects phenology, physiology, reproductive traits, and final yield at the same time, so selection based on a few traits alone may fail to identify genotypes with useful adaptive potential (Liu et al., 2023; Hossain et al., 2024). Besides, only limited research has combined trait-wise heat tolerance indices with multivariate and ideotype-based approaches to evaluate diverse rice germplasm in a breeding-oriented framework. Therefore, there is still a need for an integrated evaluation system that can identify genotypes with stable performance across multiple traits under field-based high-temperature environments and compare their responses with standard checks. In this context, the present study employed an integrated analytical framework in which trait-wise HTI was used to quantify heat tolerance responses; correlation and path coefficient analyses were employed to identify key traits associated with yield maintenance; PCA and cluster analysis were implemented to examine multivariate patterns and genetic divergence; and MGIDI-based ranking served as the principal integrative tool for selecting high-yielding and heat-tolerant rice genotypes for climate-adaptive breeding programs.

## 2 Materials and methods

### 2.1 Germplasm description

A total of 80 rice accessions were assessed in the present investigation. The germplasm was obtained from the Rice Research Institute, Tirur; Tamil Nadu Agricultural University, Coimbatore; along with collections from local farmers across India (**Table 1**). The experimental set comprised 45 traditional landraces and 35 improved cultivars. Among them, Nagina 22 and Vandana were included as heat-tolerant and susceptible checks, respectively.

**Table 1:** Rice genotypes evaluated in the present study, their classification and source of collection.

S. no	Genotypes	Classification	Source	S. no	Genotypes	Classification	Source
1	54R60	Variety	Farmer’s Field, Vellore	41	MTU 1156	Variety	TNAU, Coimbatore
2	ADT 37	Variety	Rice Research Station, Tirur	42	Mysore Malli	Landrace	TNAU, Coimbatore
3	ADT 39	Variety	Rice Research Station, Tirur	43	Nagina 22	Variety	TNAU, Coimbatore
4	ADT 46	Variety	Rice Research Station, Tirur	44	Nattu Basmati	Landrace	TNAU, Coimbatore
5	ADT 53	Variety	Rice Research Station, Tirur	45	Navara	Landrace	TNAU, Coimbatore
6	ADT 54	Variety	Rice Research Station, Tirur	46	Nei Kichadi	Landrace	TNAU, Coimbatore
7	Adukkan	Landrace	TNAU, Coimbatore	47	NLR 3238	Variety	Farmer’s Field, Nellore
8	Akashya	Variety	Farmer’s Field, Kanchipuram	48	Ottukichadi	Landrace	TNAU, Coimbatore
9	ANNA R 4	Variety	TNAU, Coimbatore	49	Padu 101	Variety	Farmer’s Field, Vellore
10	Annai Komban	Landrace	TNAU, Coimbatore	50	Pal Kudai Vazhai	Landrace	TNAU, Coimbatore
11	ASD 16	Variety	Farmer’s Field, Kanchipuram	51	Polinel	Landrace	TNAU, Coimbatore
12	ASD 37	Variety	Rice Research Station, Tirur	52	Poongar	Landrace	TNAU, Coimbatore
13	Bhavani	Landrace	TNAU, Coimbatore	53	PR 128	Variety	Farmer’s Field, Punjab
14	Bhuthakaima	Landrace	TNAU, Coimbatore	54	PR 47	Variety	Farmer’s Field, Punjab
15	Chengalpattu Sirumani	Landrace	TNAU, Coimbatore	55	Rakthasali	Landrace	TNAU, Coimbatore
16	Chinaadukunel	Landrace	TNAU, Coimbatore	56	Ramakali	Landrace	TNAU, Coimbatore
17	Chinnar	Landrace	TNAU, Coimbatore	57	RNR 15048	Variety	Farmer’s Field, Telangana
18	CO51	Variety	Rice Research Station, Tirur	58	Sahbhagi Dhan	Variety	Farmer’s Field, Odisha
19	CO54	Variety	Rice Research Station, Tirur	59	Sathayu	Variety	Farmer’s Field, Chennai
20	CO55	Variety	Rice Research Station, Tirur	60	Semballai	Landrace	TNAU, Coimbatore
21	CO56	Variety	Rice Research Station, Tirur	61	Sembuli Samba	Landrace	TNAU, Coimbatore
22	Illupaipoo Samba	Landrace	TNAU, Coimbatore	62	Sivan Samba	Landrace	TNAU, Coimbatore
23	Indhurani	Landrace	TNAU, Coimbatore	63	Sivapukavuni	Landrace	TNAU, Coimbatore
24	IR 20	Variety	Rice Research Station, Tirur	64	Thooyamalli	Landrace	TNAU, Coimbatore
25	IR 50	Variety	Rice Research Station, Tirur	65	TKM 13	Variety	Rice Research Station, Tirur
26	Kadaikaluthan	Landrace	TNAU, Coimbatore	66	TKM 15	Variety	Rice Research Station, Tirur
27	Kalluputhan	Landrace	TNAU, Coimbatore	67	TKM 9	Variety	Rice Research Station, Tirur
28	Kalurundaiyan	Landrace	TNAU, Coimbatore	68	TPS 5	Variety	TNAU, Coimbatore
29	Kamban Samba	Landrace	TNAU, Coimbatore	69	TRY 1	Variety	TNAU, Coimbatore
30	Kandhasala	Landrace	Farmer’s Field, Kerala	70	TRY 5	Variety	TNAU, Coimbatore
31	Karunkuruvai	Landrace	TNAU, Coimbatore	71	Tulasi Vasanai Seeraga Samba	Landrace	TNAU, Coimbatore
32	Karuthukar	Landrace	TNAU, Coimbatore	72	Vadakathi Samba	Landrace	TNAU, Coimbatore
33	Kattuyanam	Landrace	TNAU, Coimbatore	73	Vaigunda Red	Landrace	TNAU, Coimbatore
34	KNM 1638	Variety	Farmer’s Field, Telangana	74	Vaigunda White	Landrace	TNAU, Coimbatore
35	Kochin Samba	Landrace	TNAU, Coimbatore	75	Vallan Samba	Landrace	TNAU, Coimbatore
36	Kuliyadichan	Landrace	TNAU, Coimbatore	76	Vandana	Variety	Rice Research Station, Tirur
37	Kuthiraivalli Samba	Landrace	TNAU, Coimbatore	77	Varakal	Landrace	TNAU, Coimbatore
38	Mani Samba	Landrace	TNAU, Coimbatore	78	Vasanai Seeraga Samba	Landrace	TNAU, Coimbatore
39	Milagu Samba	Landrace	TNAU, Coimbatore	79	Vellai Kudai Vazhai	Landrace	TNAU, Coimbatore
40	MPR 606	Variety	Farmer’s Field, Vellore	80	White Ponni	Variety	TNAU, Coimbatore

### 2.2 Experimental location

The research farms of Vellore Institute of Technology (VIT) in Vellore, Tamil Nadu, India, served as testing sites for field trials, which took place during kharif 2024 and late rabi to summer 2025. The two experimental locations were Bramapuram Farm and CBMR Farm, located at VIT, Vellore. The geographic coordinates of Bramapuram Farm and CBMR Farm are 12°58′8″ N, 79°9′40″ E and 12°58′0.12″ N, 79°9′22.08″ E, respectively.

Experiments were set up at these two locations, Bramapuram and CBMR Farms, with varying sowing dates during the Kharif season (normal environment), in order to capture both temporal and geographical variability under monsoon conditions. However, during the Late Rabi–summer season (heat-stress environment) studies were carried out at Bramapuram Farm employing two staggered sowing dates to create an additional heat-stressed environment. The Summer studies were intended to expose genotypes to higher temperatures during the reproductive stage, whereas the Kharif trials aimed to assess genotypic performance under comparatively moderate climatic conditions.

In total, the study comprised four field environments. Two normal environment trials were conducted during 2024, with sowing on 7 June 2024 at Bramapuram Farm (Normal Trial I) and 20 June 2024 at CBMR Farm (Normal Trial II). Two heat stress trials were conducted during 2025 at Bramapuram Farm, with sowing on 5 January 2025 (Heat Stress Trial I) and 10 February 2025 (Heat Stress Trial II). In the Vellore region, the late rabi-summer period is naturally characterized by high temperature, particularly from March to June, which increases the probability of exposing the reproductive stage to heat stress. Therefore, the kharif trials represented relatively moderate growing conditions, whereas the late rabi-summer trials were used to evaluate genotypic response under naturally elevated temperature conditions. However, the two stress environments differed in the magnitude and pattern of thermal exposure.

### 2.3 Experimental design and agronomic practices

The experiment was conducted using an alpha-lattice design with three biological replicates. Each accession was grown in separate plots comprising two rows, each 4 m in length. Spacing of 20 × 20 cm was maintained between rows and plants, respectively, with each row accommodating 20 plants, resulting in approximately 40 plants per plot. Thus, the effective plot size was 1.6 m^2^. Observations on yield and yield-associated characters were observed from three randomly selected plants from the middle of each plot in every replication, excluding border plants to minimize edge effects. Mean values were used for subsequent statistical analyses. Standard organic crop management practices recommended by Tamil Nadu Agricultural University were followed under both normal and heat-stressed environments. Particularly, bone meal, vermicompost, neem cake, and farmyard manure were utilized to increase soil fertility and guarantee the best possible crop growth.

### 2.4 Weather parameters and stress imposition

Throughout the trial period, meteorological data were captured by the observatory at VIT School of Agricultural Innovations and Advanced Learning (VAIAL), Sevoor Farm. Weekly minimum temperatures varied between 16.5°C and 25.9°C, whereas weekly maximum temperatures under the normal environment (June 2024–December 2024) ranged from 28.8°C to 36.9°C. With sufficient rainfall (1549.9 mm) and sunshine, the afternoon relative humidity (14:22h) was often kept between 40% and 60%, providing ideal conditions for grain filling and flowering. On the other hand, genotypes were subjected to noticeably higher temperatures during the reproductive stage in the heat-stress environment (December 2024–July 2025). While low temperatures varied from 22°C to 27°C, maximum temperatures were often above 35°C, peaking at 37°C–40°C. Relative humidity dropped to 33%–45% in the afternoon, and there was low rainfall (378.7 mm) and 7–9h of sunshine. These conditions imposed temperatures that were 4°C–6°C during the reproductive stage compared to the normal environment.

### 2.5 Trait measurement

#### 2.5.1 Phenological and morphological traits

The observations on yield and yield-associated traits were recorded from three randomly selected plants from each replication, and the mean values were used for statistical analysis. Days to first flowering (DF) and days to 50% flowering (DFF) were recorded through daily field observations from sowing until flowering. Plant height (PH), number of productive tillers (PT), panicle length (PL), flag leaf length (FLL), flag leaf breadth (FLB), and root length (RL) were measured at appropriate growth stages using the Standard Evaluation System (SES) for rice (IRRI, 2013).

#### 2.5.2 Physiological and derived traits

##### 2.5.2.1 SPAD

Relative chlorophyll content was measured using a SPAD-502 meter (Konica Minolta, Japan). Readings were recorded from the middle portion of fully expanded flag leaves during the reproductive stage between 10:00 and 11:00 h to minimize diurnal variability (Devi et al., 2023).

##### 2.5.2.2 Leaf temperature

Leaf temperature was recorded by using an infrared thermometer during peak stress hours (12:00-14:00 h) (Chen, 2015).

##### 2.5.2.3 Moisture content

Moisture content of above-ground (MCA) and below-ground (MCB) tissues were determined using the oven-drying method. The fresh weight of shoot and root samples was recorded immediately after sampling. For dry weight measurement, samples were oven dried at 70°C until no further change in weight was observed (Zhou et al., 2021). Moisture content was determined according to the equation below Equation 1.

(1) $$MC=\frac{Fresh\;weight-Dry\;weight}{Fresh\;weight}\times 100\;$$

##### 2.5.2.4 Root-shoot ratio

Root-shoot ratio was calculated on a dry weight basis to assess biomass allocation under stress conditions (Lopez et al., 2023). RSR was computed using the Equation 2:

(2) $$RSR=\frac{Dry\;root\;weight}{Dry\;shoot\;weight}$$

#### 2.5.3 Reproductive and yield traits

Number of grains per panicle (NGPP), number of filled grains per panicle (NFGP), and spikelet fertility (SF) were recorded at maturity. Hundred-grain weight (HGW), panicle weight (PW), and single plant yield (SPY) were noted after drying grains to 14% moisture content.

##### 2.5.3.1 Spikelet fertility (%)

The spikelet fertility (SF) was calculated using the formula with the number of filled grains per panicle and the total number of grains per panicle as follows Equation 3:

(3) $$Sf=\frac{Number\;of\;filled\;grains\;per\;panicle\;}{Number\;of\;grains\;per\;panicle}\;\times 100\;$$

##### 2.5.3.2 Harvest index 

The harvest index (HI) was calculated using grain yield and total dry biological yield for each genotype and expressed as a percentage. The mean grain yield, dry biological yield and harvest index were given in the **Supplementary Table 7**, Equation 4.

(4) $$HI=\frac{Grain\;Yield}{Total\;biological\;yield}\;\times 100$$

The reproductive and yield traits were taken using the Standard Evaluation System (SES) for rice (IRRI, 2013).

### 2.6 Heat tolerance index computation

The HTI was computed using Microsoft Excel (version 2511) based on trait data recorded under both normal and heat-stress environments. For each trait, the mean performance of individual genotypes under non-stress conditions (Xp) and stress conditions (Xs) was obtained by pooling data across replications within the respective environments. In addition, the overall mean response of all genotypes under non-stress conditions ( $\bar{Xp}$)^2^ was computed. HTI was estimated for all recorded agro-morphological and physiological traits to evaluate trait-specific tolerance under elevated temperature conditions. The calculated HTI values were used for all statistical analyses. The index was calculated using the Stress Tolerance Index (STI) proposed by Fernandez (1992). As the present investigation centered on heat stress, the index was termed the HTI Equation 5.

(5) $$HTI=\frac{\left(Xp\times Xs\right)}{{\left(\bar{Xp}\right)}^{2}}$$

HTI was calculated trait-wise and interpreted according to the biological desirability of each trait under heat stress. For favorable traits such as single plant yield, panicle weight, spikelet fertility, harvest index, number of grains per panicle, and number of filled grains per panicle, higher HTI values were considered desirable. For traits such as leaf temperature and delayed flowering, lower values were considered biologically favorable under stress; therefore, HTI values were interpreted in relation to stress response rather than direct superiority. In general, HTI values greater than 1 indicated better relative performance under heat stress, whereas values below 1 indicated greater reduction under stress conditions. The interpretation was made in relation to the biological desirability of each trait.

### 2.7 Data analysis and multivariate approaches

R software (version 4.3.2) was used for pooled analysis of variance (ANOVA) to investigate the impact of genotype, environment, and genotype × environment interaction in an alpha-lattice design. Replications and blocks inside replications were treated as random effects. The ‘agricolae’ software was used to perform ANOVA computations.

Correlation analysis among HTI values and component traits was conducted using the ‘corrplot’ package in R to determine interrelationships under heat stress. Principal component analysis (PCA) was applied using the ‘FactoMineR’ and ‘factoextra’ packages used to investigate multivariate relationships among genotypes based on heat tolerance responses. Genetic diversity among genotypes was assessed through hierarchical clustering using Mahalanobis D² statistics, and clustering was performed using Ward’s minimum variance method implemented in the ‘stats’ package of R. Inter-cluster and intra-cluster distances were calculated, and circular dendrograms were generated and visualized using iTOL (version 6.9). The Multi-Trait Genotype-Ideotype Distance Index (MGIDI) (Olivoto and Lúcio, 2020) was employed to identify superior genotypes based on their distance from an ideal ideotype while simultaneously considering multiple traits. MGIDI analysis was conducted using the “metan” package in R, utilizing HTI-based values. Trait desirability (increase/decrease) was defined according to breeding objectives and adopting a selection intensity of 15%. Selection gain (%) in the MGIDI analysis was calculated as follows Equation 6:

(6) $$SG\%=[\frac{Xs-Xo}{Xo}]\times 100$$

Where Xs represents the mean of selected genotypes, and Xo represents the overall population mean, both calculated from the HTI trait dataset (Olivoto and Nardino, 2021).

### 2.8 Flowering window thermal characterization

To quantify genotype-specific reproductive stage heat exposure, flowering window-based thermal summaries were generated for each genotype in each environment. The flowering window was considered the interval between the mean DF and the mean DFF. Using daily weather records, the corresponding mean maximum temperature, mean minimum temperature, number of days with maximum temperature above 35°C, flowering window duration and heat degree days (HDD) were extracted for each genotype-environment combination. In addition, HDD values were calculated to quantify cumulative heat load during the flowering window as follows Equation 7:

(7) $$HDD=\sum_{i=DF}^{DFF}\;(T_{max,i}-35),\;\text{ for }T_{max,i}>35\text{°C}$$

Where $T_{max,i}$ was the daily maximum temperature on day i during the flowering window, and for days with $T_{max,i}$ < 35 °C, the contribution was taken as zero. The equation (6) was adapted from the heat degree days approach described in Liu et al. (2014). These summaries were presented in **Supplementary Table 5** and **Supplementary Figure 1**. For group-wise comparison, genotypes were classified into early, intermediate and late-flowering groups based on pooled normal-environment DFF. Genotypes with a mean DFF < 95.5 days were classified as early flowering, those with a DFF between 95.5 and 103 days as intermediate flowering, and those with a DFF greater than 103 days as late flowering (**Supplementary Table 6**). The resulting flowering group-wise thermal summary is presented in **Table 2**.

**Table 2:** Flowering group-wise thermal summary across normal and heat-stress environments.

Flowering group	Condition	Avg mean Tmax	Avg mean Tmin	Avg days above 35°C	Number of genotypes
Early flowering	Normal Trial I	35.28	25.35	7	29
Early flowering	Normal Trial II	35.01	24.89	5	29
Early flowering	Heat stress Trial I	36.88	25.06	12	29
Early flowering	Heat stress Trial II	35.87	26.33	8	29
Intermediate flowering	Normal Trial I	34.97	24.65	4	25
Intermediate flowering	Normal Trial II	35.05	25.07	6	25
Intermediate flowering	Heat stress Trial I	37.06	25.26	10	25
Intermediate flowering	Heat stress Trial II	35.44	26.56	7	25
Late flowering	Normal Trial I	35.10	24.85	5	26
Late flowering	Normal Trial II	35.29	24.13	7	26
Late flowering	Heat stress Trial I	36.90	25.00	13	26
Late flowering	Heat stress Trial II	35.53	26.26	8	26

## 3 Results

### 3.1 Analysis of variance

Pooled analysis of variance demonstrated highly significant effects of genotype, environment, and genotype × environment interaction (*p* < 0.001) for all studied characters (**Table 3**), reflecting substantial genetic variability and pronounced differential responses of genotypes across temperature regimes. Replication and block effects were non-significant, indicating effective control of experimental variability under the alpha-lattice design.

**Table 3:** Pooled analysis of variance for 20 traits of rice genotypes evaluated under normal and heat stress environments.

Source of variation		Mean sum of squares									
	Df	DF	DFF	PH	PT	PL	FLB	FLL	RL	HGW	SPY
Genotypes	79	574.39**	739.76**	6251.02**	284.83 **	122.46 **	0.44**	302.77**	240.68**	3.36**	336.70**
Environment	1	45858.22**	49061.01**	30937.04**	19023.57**	1434.26**	2.88**	2522.60**	3481.62**	99.29**	59772.24**
Genotypes: Env	79	455.48**	537.13**	421.74**	62.03**	11.86**	0.09**	23.96**	35.15**	2.26**	193.48**
Env: Replication	4	2.45 ns	3.89 ns	7.90 ns	1.12 ns	1.01 ns	0.01 ns	0.22 ns	0.29 ns	0.18 ns	1.44 ns
Replication: Block	42	36.27 ns	34.16 ns	203.32 ns	27.90 ns	5.05 ns	0.02 ns	8.90 ns	8.98 ns	0.18 ns	14.56 ns
Residuals	754	40.01	46.20	250.90	41.76	4.17	0.02	10.55	9.30	0.20	15.19
Source of variation		Mean sum of squares									
		PW	SPAD	LT	NGPP	NFGP	SF	MCA	MCB	HI	RSR
Genotypes	79	5.41**	68.57**	18.10**	17488.67**	15559.50**	1495.69**	1635.77 **	1860.70**	913.23**	0.90**
Environment	1	507.14**	305.02**	5434.68**	163922.38**	700631.32**	175890.11**	26240.48**	29911.15**	661.23**	2.00**
Genotypes: Env	79	4.19**	47.26**	28.00**	3711.01**	6434.51**	1352.01**	399.07**	456.75**	236.43**	0.20**
Env: Replication	4	0.04 ns	5.11 ns	1.64 ns	13.46 ns	10.80 ns	3.81 ns	102.85 ns	9.45 ns	6.32 ns	0.00 ns
Replication: Block	42	0.18 ns	6.05 ns	5.09 ns	117.58 ns	135.52 ns	35.25 ns	257.54 ns	145.23 ns	53.96 ns	0.06 ns
Residuals	754	0.19	8.45	5.63	130.97	144.02	66.71	330.88	202.77	105.61	0.10

ns, non-significant; **(*p* < 0.01).

DF, days to first flowering; DFF, days to 50% flowering; PH, plant height; PT, number of productive tillers; PL, panicle length; FLB, flag leaf breadth; FLL, flag leaf length; RL, root length; HGW, hundred grain weight; SPY, single plant yield; PW, panicle weight; SPAD, chlorophyll content; LT, leaf temperature; NGPP, number of grains per panicle; NFGP, number of filled grains per panicle; SF, Spikelet fertility; MCA, moisture content above biomass (Shoot); MCB, moisture content below biomass (root); HI, harvest index; RSR, root shoot ratio.

### 3.2 Trait response to heat stress

**Figure 1:**
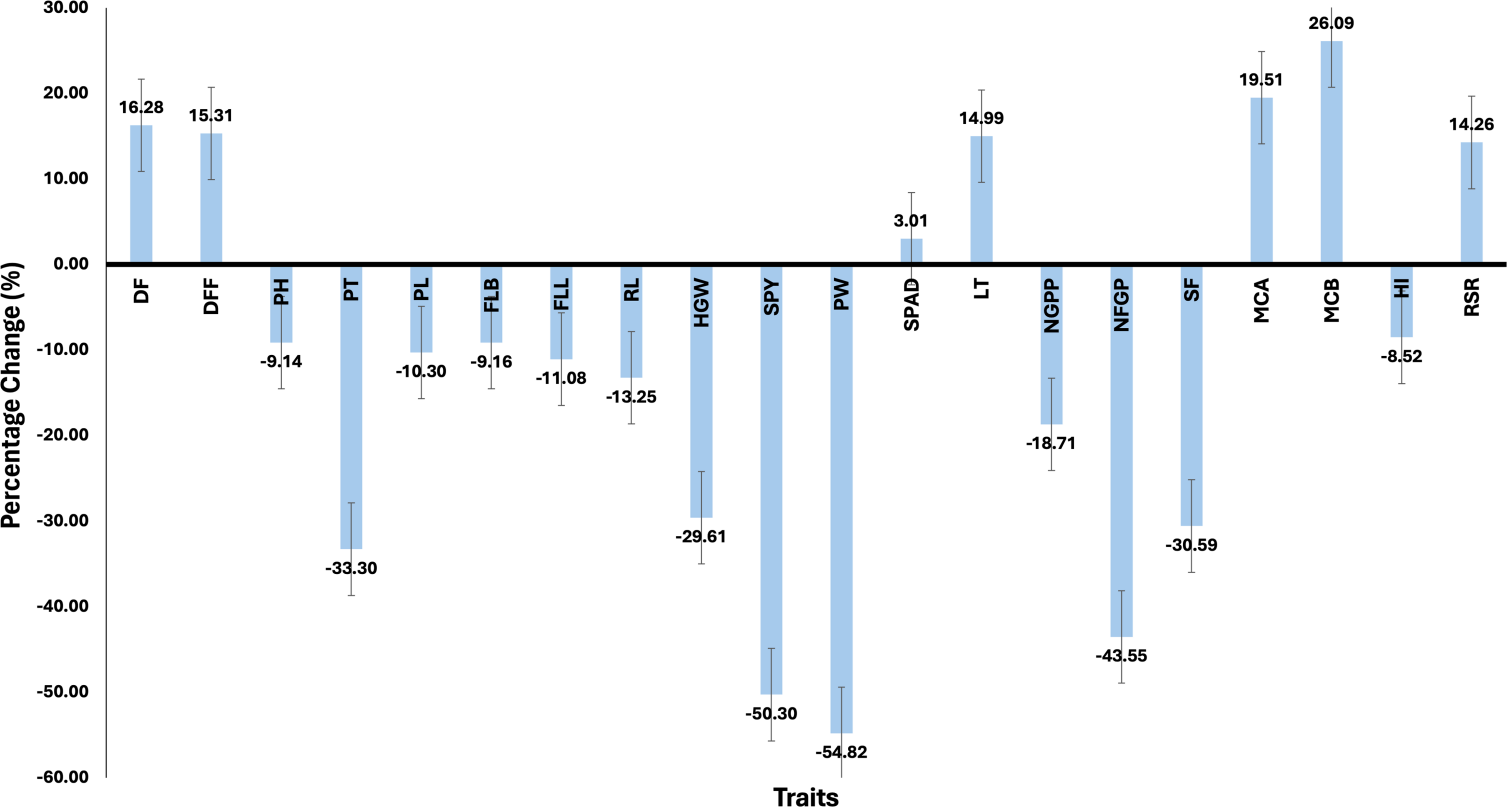
Percentage change in phenological, morphological, physiological, reproductive and yield-related traits of rice genotypes under heat stress relative to the normal environment. Positive values indicate an increase under stress, while negative values indicate a reduction.

Exposure to heat stress during the reproductive stage resulted in major changes across all studied traits, which are presented in **Table 4** and **Figure 1**. Heat stress conditions caused a delay in flowering, which resulted in first flowering (DF) and 50% flowering (DFF) times increasing by 16.28% and 15.31%, respectively, when compared to normal conditions. The majority of physical characteristics showed moderate reductions under heat stress. The study found that plant height (PH) reduced by 9.14%, panicle length (PL) decreased by 10.30%, the flag leaf width (FLB) and flag leaf length (FLL) declined by 9.16% and 11.08%, respectively, the root length (RL) reduced by 13.25%, and the productive tillers (PT) count decreased by 33.30%. Heat stress had the greatest impact on reproductive traits and yield-related characteristics. The panicle weight (PW) and single plant yield (SPY) experienced a 54.82% and 50.30% decline, respectively. The NFGP dropped by 43.55%, and spikelet fertility decreased by 30.59%. The hundred-grain weight (HGW) decreased by 29.61%, and the NGPP dropped by 18.71%. The harvest index (HI) experienced a decline of 8.52%, which represented a minor decrease. Plants showed physiological responses that matched canopy warming effects and biomass distribution changes during stress conditions. The leaf temperature (LT) experienced a 14.99% increase, while moisture content above ground (MCA) and below ground (MCB) showed increases of 19.51% and 26.09%, respectively. The root-shoot ratio (RSR) increased by 14.26%, while SPAD values showed only a 3.01% increase.

**Table 4:** Pooled mean performance and percentage change of studied traits under normal and heat-stress environments.

Traits	Pooled normal mean	Pooled stress mean	Percentage change (%)
DF (days)	86.00	100.00	16.28
DFF (days)	98.00	113.00	15.31
PH (cm)	122.65	111.44	−9.14
PT (number per plant)	26.20	17.48	−33.30
PL (cm)	23.94	21.47	−10.30
FLB (cm)	1.19	1.08	−9.16
FLL (cm)	28.26	25.13	−11.08
RL (cm)	28.68	24.88	−13.25
HGW (g)	2.16	1.52	−29.61
SPY (g)	31.26	15.54	−50.30
PW (g)	2.65	1.20	−54.82
SPAD	40.39	41.61	3.01
LT (°C)	31.77	36.53	14.99
NGPP (number per plant)	139.00	113.00	−18.71
NFGP (number per plant)	124.00	70.00	−43.55
SF (%)	87.93	61.04	−30.59
MCA (%)	52.25	62.44	19.51
MCB (%)	41.87	52.79	26.09
HI (%)	20.66	18.90	−8.52
RSR	0.65	0.75	14.26

For abbreviation check **Table 3**.

### 3.3 Heat tolerance index analysis

**Figure 2:**
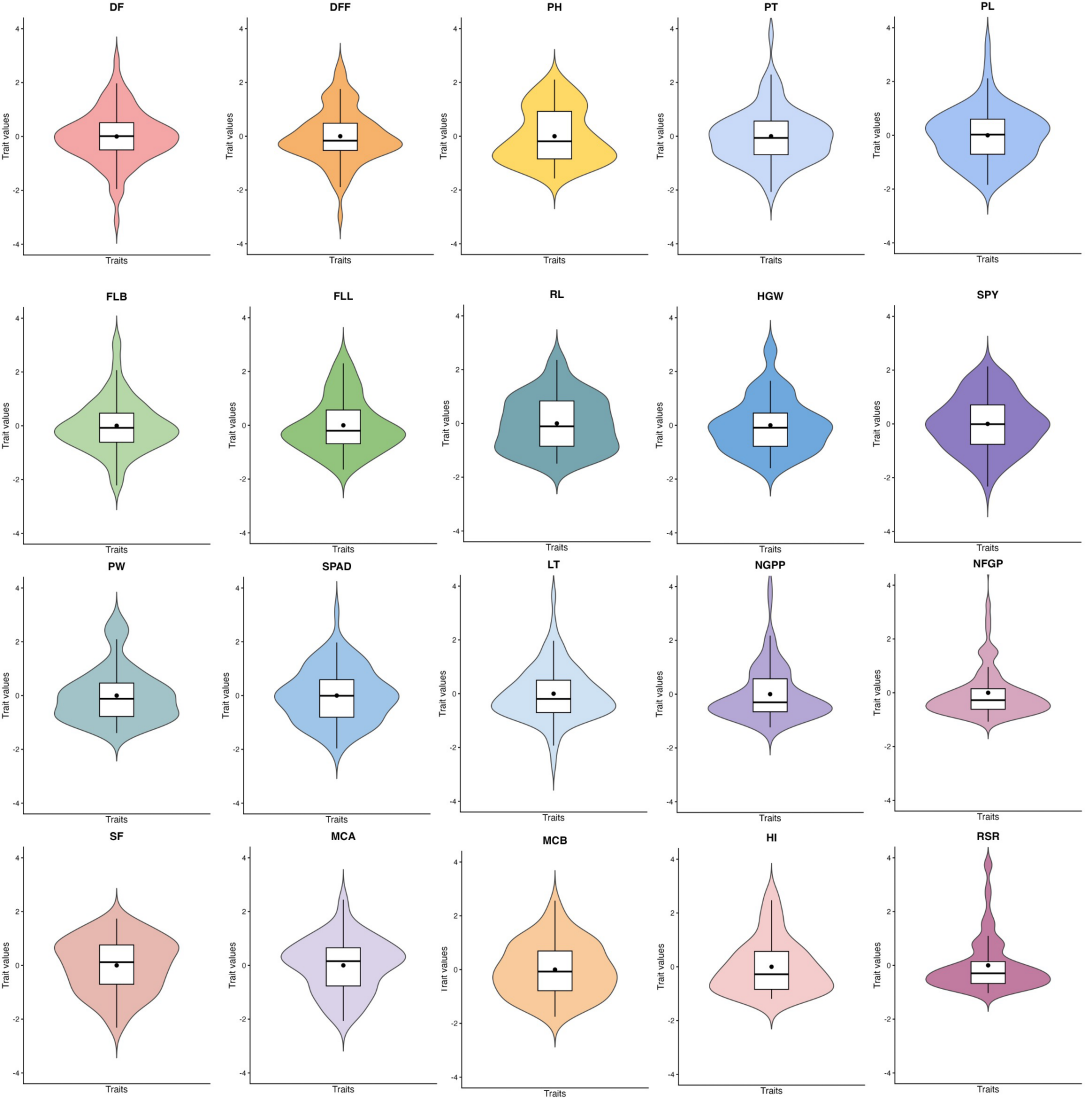
Violin plots showing the distribution of HTI mean values across rice genotypes and studied traits. The plots illustrate the density, spread and variability of heat tolerance responses.

The HTI was calculated by utilizing the pooled mean values of each genotype under normal and heat stress environments to assess stress tolerance efficiency by integrating absolute yield potential and relative stress performance. The findings demonstrated that there was significant diversity across the 20 attributes assessed in 80 genotypes (**Table 5**). HTI values for yield-related variables, such as single plant yield (0.51), panicle weight (0.47), productive tillers (0.69), number of filled grains per panicle (0.61), and spikelet fertility (0.69), were relatively lower. On the other hand, HTI values greater than 1.0 were recorded for physiological characteristics such as moisture content above ground (1.24), moisture content below ground (1.33), root-shoot ratio (1.27), leaf temperature (1.15), and SPAD value (1.03). HTI values above unity were also seen in phenological features, with values of 1.16 and 1.15 recorded for DF and DFF, respectively. There was significant genetic heterogeneity for yield stability under heat stress, as evidenced by genotype-wise HTI values for single plant yield ranging from 0.04 to 0.93. The TKM 9 (0.93), TRY 1 (0.86), PR 128 (0.85), TPS 5 (0.84), and TRY 5 (0.83) HTI values were the highest. However, the HTI values were lowest in Kandhasala (0.04), Mysore Malli (0.12), Illupaipoo Samba (0.15), and Tulasi Vasanai Seeraga Samba (0.18). HTI values for single plant yield were below 0.70 for most genotypes. HTI values for all the genotypes across all the traits were presented in a table (**Supplementary Table 1**). The wide variation in HTI mean values across genotypes and traits was further illustrated by the violin plot distribution (**Figure 2**). HTI values were interpreted according to the biological desirability of each trait, since higher values were not necessarily favorable for all characters.

**Table 5:** Descriptive statistics of HTI-based trait values in rice genotypes.

Traits	Mean	Minimum	Maximum	Standard error	Standard deviation
DF (Days)	1.16	0.63	1.64	0.02	0.17
DFF (Days)	1.15	0.65	1.58	0.02	0.17
PH (cm)	0.94	0.36	1.71	0.04	0.37
PT (Number per plant)	0.69	0.06	1.83	0.03	0.30
PL (cm)	0.91	0.44	1.79	0.03	0.25
FLB (cm)	0.93	0.26	1.89	0.03	0.30
FLL (cm)	0.91	0.35	1.80	0.04	0.34
RL (cm)	0.89	0.45	1.58	0.03	0.29
HGW (g)	0.72	0.11	1.81	0.04	0.38
SPY (g)	0.51	0.04	0.93	0.02	0.20
PW (g)	0.47	0.06	1.28	0.03	0.29
SPAD	1.03	0.79	1.41	0.01	0.12
LT (°C)	1.15	0.93	1.45	0.01	0.08
NGPP (number per plant)	0.87	0.20	3.07	0.06	0.54
NFGP (number per plant)	0.61	0.05	3.08	0.06	0.52
SF (%)	0.69	0.12	1.13	0.03	0.25
MCA (%)	1.24	0.26	2.39	0.05	0.47
MCB (%)	1.33	0.15	3.06	0.08	0.67
HI (%)	1.05	0.04	3.35	0.09	0.85
RSR	1.27	0.17	5.33	0.12	1.07

For abbreviation check **Table 3**.

### 3.4 Correlation analysis

**Figure 3:**
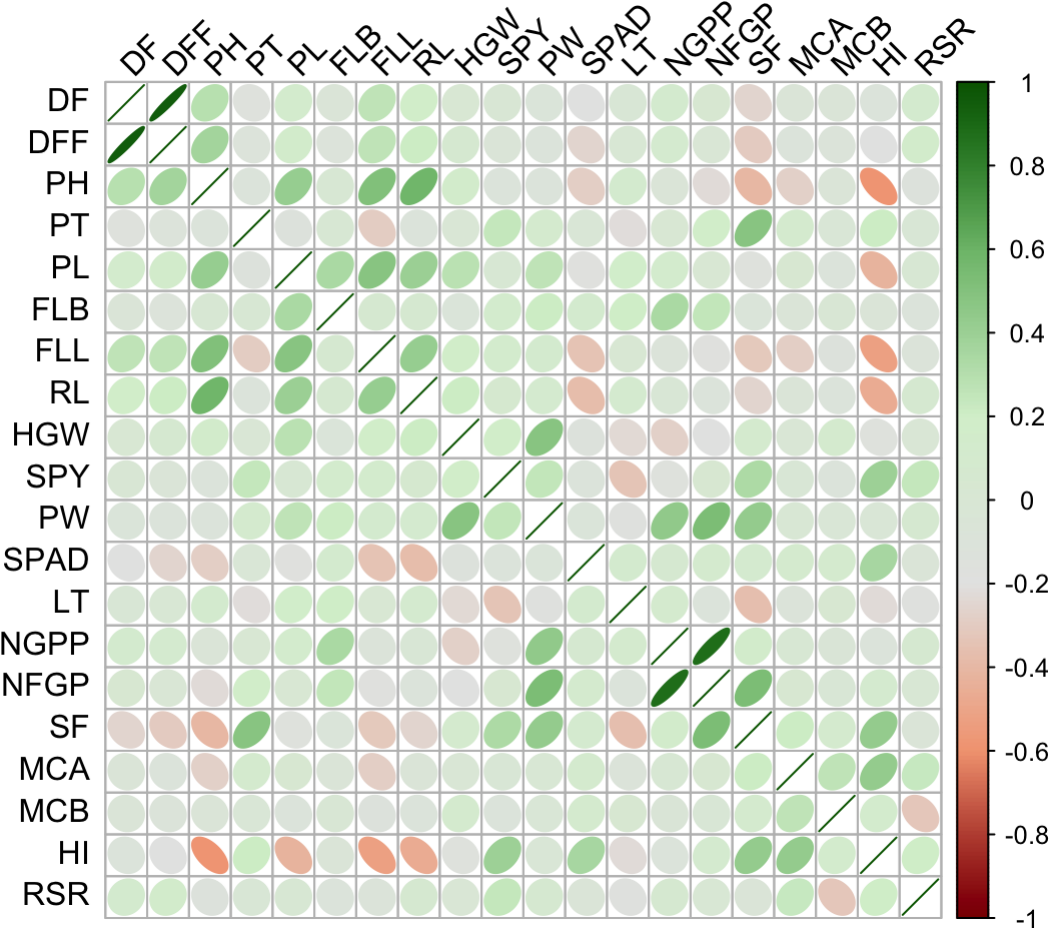
Genotypic correlation matrix of HTI-based studied traits. Green and red colors indicate positive and negative correlations, respectively, while color intensity and ellipse shape represent the magnitude of association.

The correlation analysis was carried out using HTI-based trait values to explain the pattern of association among the traits related to heat tolerance response. The results revealed multiple significant associations among the studied traits. A very strong and positive correlation was observed between DF and DFF (0.96**). PH was positively correlated with FLL (0.52**), root length (0.58**), and panicle length (0.43**), whereas it was negatively correlated with harvest index (−0.59**). SPY exhibited positive relationships with the PT (0.25*), panicle weight (0.26*), spikelet fertility (0.34*), and harvest index (0.39**). In contrast, leaf temperature showed a negative association with yield (−0.33*). Panicle weight was positively correlated with NGPP (0.45**) and the NFGP (0.53**). Similarly, NGPP and NFGP demonstrated a strong positive correlation (0.89**). Spikelet fertility was negatively correlated with PH (−0.41**), DFF (−0.31*), and leaf temperature (−0.36**), while positive correlations were recorded with the PT (0.49**), panicle weight (0.43**), and harvest index (0.44**). In addition, SPAD showed negative correlations with flag leaf length (−0.35*) and root length (−0.38**). The harvest index was positively correlated with SPY (0.39**), spikelet fertility (0.44**), and MCA (0.43**). Furthermore, root-to-shoot ratio was positively associated with yield (0.24*) and MCA (0.24*), but negatively associated with MCB (−0.33*). The correlation plot is shown in **Figure 3**.

### 3.5 Path coefficient analysis

Genotypic path coefficient analysis based on HTI values is presented in **Table 6**. Harvest index (HI) exhibited the strongest positive direct effect on SPY by (0.629), indicating its major contribution to yield under heat stress. The NFGP and the NGPP both produced significant direct impacts that resulted in positive yield effects. Flag leaf length showed a direct effect of 0.287 on yield. Positive direct effects were also observed for RSR (0.223), FLB (0.203), DF (0.194), PW (0.155), RL (0.153), and PT (0.146). The days to 50% flowering showed a direct negative impact on yield, which was measured with an effect of −0.241. Negative direct effects were also recorded for SPAD (−0.141), LT (−0.076), and MCA (−0.234), whereas the direct effects of MCB and spikelet fertility were negligible. Plant height created a small positive direct effect, but it created a major negative indirect effect through harvest index, which was estimated at −0.371. The positive genotypic correlations of yield with HI (0.390), SF (0.337), PW (0.256), PT (0.248), and RSR (0.244) were mainly attributed to their substantial direct effects, along with supportive indirect contributions through other traits. The residual effect (0.6326) indicated that a moderate proportion of variation in yield remained unexplained by the traits included in the analysis.

**Table 6:** Direct and indirect effects of HTI-based traits on single-plant yield estimated by genotypic path coefficient analysis.

Trait	DF	DFF	PH	PT	PL	FLB	FLL	RL	HGW		PW	SPAD	LT	NGPP	NFGP	SF	MCA	MCB	HI	RSR	rg_with_Yield
DF	0.194	−0.231	0.016	−0.021	0.003	−0.012	0.075	0.028	0.001	−0.012		0.027	0.001	−0.058	0.014	−0.001	0.015	0.000	−0.067	0.026	-0.002
DFF	0.186	−0.241	0.020	−0.017	0.004	−0.024	0.076	0.033	0.002	−0.014		0.036	0.000	−0.042	−0.009	−0.001	0.023	0.000	−0.115	0.032	-0.051
PH	0.057	−0.090	0.054	−0.015	0.012	0.005	0.149	0.089	0.005	−0.017		0.040	−0.009	0.032	−0.080	−0.002	0.065	0.000	−0.371	−0.029	−0.104
PT	−0.027	0.029	−0.005	0.146	−0.004	0.005	−0.083	−0.014	−0.001	0.014		0.005	0.016	−0.010	0.069	0.002	−0.023	0.000	0.132	−0.004	0.248*
PL	0.024	−0.036	0.023	−0.019	0.028	0.069	0.139	0.063	0.009	0.041		0.025	−0.013	−0.066	0.005	−0.001	−0.008	0.000	−0.267	0.005	0.021
FLB	−0.012	0.028	0.001	0.003	0.009	0.203	0.018	0.010	−0.003	0.034		−0.016	−0.016	−0.180	0.092	0.000	0.014	0.000	−0.035	-0.021	0.131
FLL	0.051	−0.064	0.028	−0.042	0.013	0.013	0.287	0.066	0.006	0.018		0.049	−0.001	0.046	−0.065	−0.001	0.067	0.000	−0.330	-0.018	0.122
RL	0.036	−0.052	0.032	−0.014	0.011	0.014	0.123	0.153	0.007	0.013		0.053	−0.007	0.010	−0.037	−0.001	0.016	0.000	−0.291	0.013	0.079
HGW	0.004	−0.017	0.008	−0.004	0.008	−0.016	0.055	0.033	0.032	0.075		0.018	0.018	0.144	−0.072	0.000	0.006	0.000	−0.096	-0.006	0.191
PW	−0.015	0.022	−0.006	0.014	0.007	0.044	0.033	0.013	0.015	0.155		0.011	0.012	−0.232	0.193	0.002	-0.001	0.000	−0.024	0.013	0.256*
SPAD	−0.037	0.062	−0.015	−0.006	−0.005	0.023	−0.100	−0.058	−0.004	−0.012		−0.141	−0.009	−0.027	0.037	0.000	−0.022	0.000	0.222	−0.015	−0.105
LT	−0.002	−0.001	0.006	−0.031	0.005	0.041	0.005	0.013	−0.007	−0.025		−0.016	−0.076	−0.052	−0.034	−0.001	0.019	0.000	−0.139	−0.036	−0.332*
NGPP	0.022	−0.020	−0.003	0.003	0.004	0.071	−0.025	−0.003	−0.009	0.069		−0.007	−0.008	−0.518	0.322	0.001	−0.006	0.000	−0.060	0.014	−0.154
NFGP	0.007	0.006	−0.012	0.028	0.000	0.051	−0.052	−0.016	−0.006	0.082		−0.014	0.007	−0.459	0.363	0.002	−0.008	0.000	0.055	0.002	0.037
SF	−0.049	0.074	−0.022	0.072	−0.004	−0.016	−0.091	−0.040	0.003	0.067		−0.012	0.028	−0.082	0.193	0.004	−0.051	0.000	0.277	−0.014	0.337*
MCA	−0.013	0.023	−0.015	0.014	0.001	−0.012	−0.083	−0.010	−0.001	0.000		−0.013	0.006	−0.013	0.013	0.001	−0.234	−0.001	0.271	0.053	−0.013
MCB	−0.011	0.020	−0.003	−0.006	−0.003	0.000	−0.035	−0.014	0.003	−0.006		−0.015	−0.003	0.024	−0.006	0.000	−0.061	−0.003	0.082	−0.073	−0.106
HI	−0.021	0.044	−0.032	0.031	−0.012	−0.011	−0.151	−0.071	−0.005	−0.006		−0.050	0.017	0.049	0.032	0.002	−0.101	0.000	0.629	0.046	0.390**
RSR	0.023	−0.035	−0.007	−0.002	0.001	−0.019	−0.024	0.009	−0.001	0.009		0.009	0.012	−0.033	0.004	0.000	−0.055	0.001	0.130	0.223	0.244*

Residual effect = 0.6236; ns, non-significant; *p < 0.05; and **p < 0.01; For abbreviation check **Table 3**.

### 3.6 Principal component analysis

The PCA analysis performed under heat stress conditions generated eight principal components with eigenvalues greater than 1.0, explaining 79.17% of the total variability across 20 studied traits. The results of the PCA analysis are given in **Table 7**. The individual contributions of each principal component were 20.92%, 13.64%, 11.04%, 9.65%, 6.83%, 6.54%, 5.33%, and 5.23%, respectively. The analysis of PC1 factor loadings showed that PH, FLL, RL, PL, DF, and DFF produced strong negative effects, while harvest index and spikelet fertility generated positive effects that exceeded their negative impacts. The component explained most of the variation that occurred in plants during their vegetative and phenological development. PC2 was categorized by high loadings for PW, NFGP, and NGPP, suggesting its association with grain productivity and reproductive performance. The positive influence of PC3 originated from leaf temperature, while SPY and HSW had a greater influence through negative loadings. The phenological changes that occurred between different genotypes were displayed through days to flowering and days to 50% flowering, which established PC4 as the main governing factor. PC5 established root-to-shoot ratio as its primary positive element, while MCB produced strong negative effects. PC6 was primarily associated with MCA, while PC7 had productive tillers as its main component. PC8 received its main contributions from flag leaf breadth and single plant-yield measurements.

**Table 7:** Eigenvalues, variance contribution, cumulative variance, and trait loadings of principal components based on HTI values.

Components	PC1	PC2	PC 3	PC 4	PC 5	PC 6	PC 7	PC 8
Eigenvalue	4.18	2.73	2.21	1.93	1.37	1.31	1.07	1.05
Variance percentage	20.92	13.64	11.04	9.65	6.83	6.54	5.33	5.23
Cumulative percentage	20.92	34.56	45.60	55.24	62.07	68.61	73.94	79.17
DF	−0.23	0.07	−0.06	−0.55	−0.24	0.03	0.11	0.22
DFF	−0.27	0.06	−0.08	−0.53	−0.23	0.00	0.07	0.15
PH	−0.38	0.04	−0.02	0.06	−0.07	−0.08	−0.23	0.10
PT	0.19	0.14	−0.17	0.01	−0.01	−0.29	−0.53	0.25
PL	−0.26	0.26	0.02	0.18	0.07	0.35	−0.09	0.04
FLB	−0.02	0.24	0.27	0.15	0.21	0.25	−0.02	0.52
FLL	−0.35	0.12	−0.10	0.12	0.04	−0.02	0.14	0.09
RL	−0.31	0.15	−0.11	0.09	0.05	0.10	−0.35	−0.08
HGW	−0.10	0.15	−0.37	0.26	−0.27	0.16	0.33	−0.12
SPY	0.09	0.20	−0.41	0.01	0.25	0.06	0.05	0.46
PW	0.06	0.49	−0.07	0.15	−0.08	0.06	0.28	−0.17
SPAD	0.21	−0.12	0.23	0.03	0.00	0.21	0.34	0.23
LT	−0.12	−0.12	0.41	0.08	0.07	0.20	−0.09	0.10
NGPP	0.06	0.41	0.40	−0.19	−0.01	−0.03	−0.03	−0.15
NFGP	0.18	0.46	0.26	−0.16	−0.08	−0.14	−0.01	−0.08
SF	0.33	0.27	−0.17	0.07	−0.18	−0.16	−0.10	0.00
MCA	0.18	−0.01	−0.08	−0.11	−0.15	0.58	−0.39	−0.23
MCB	0.08	−0.08	0.05	0.14	−0.64	0.25	−0.12	0.14
HI	0.37	−0.07	−0.16	−0.19	0.03	0.23	0.04	0.24
RSR	0.03	0.07	−0.20	−0.32	0.46	0.30	−0.01	−0.31

For abbreviation check **Table 3**.

**Figure 4:**
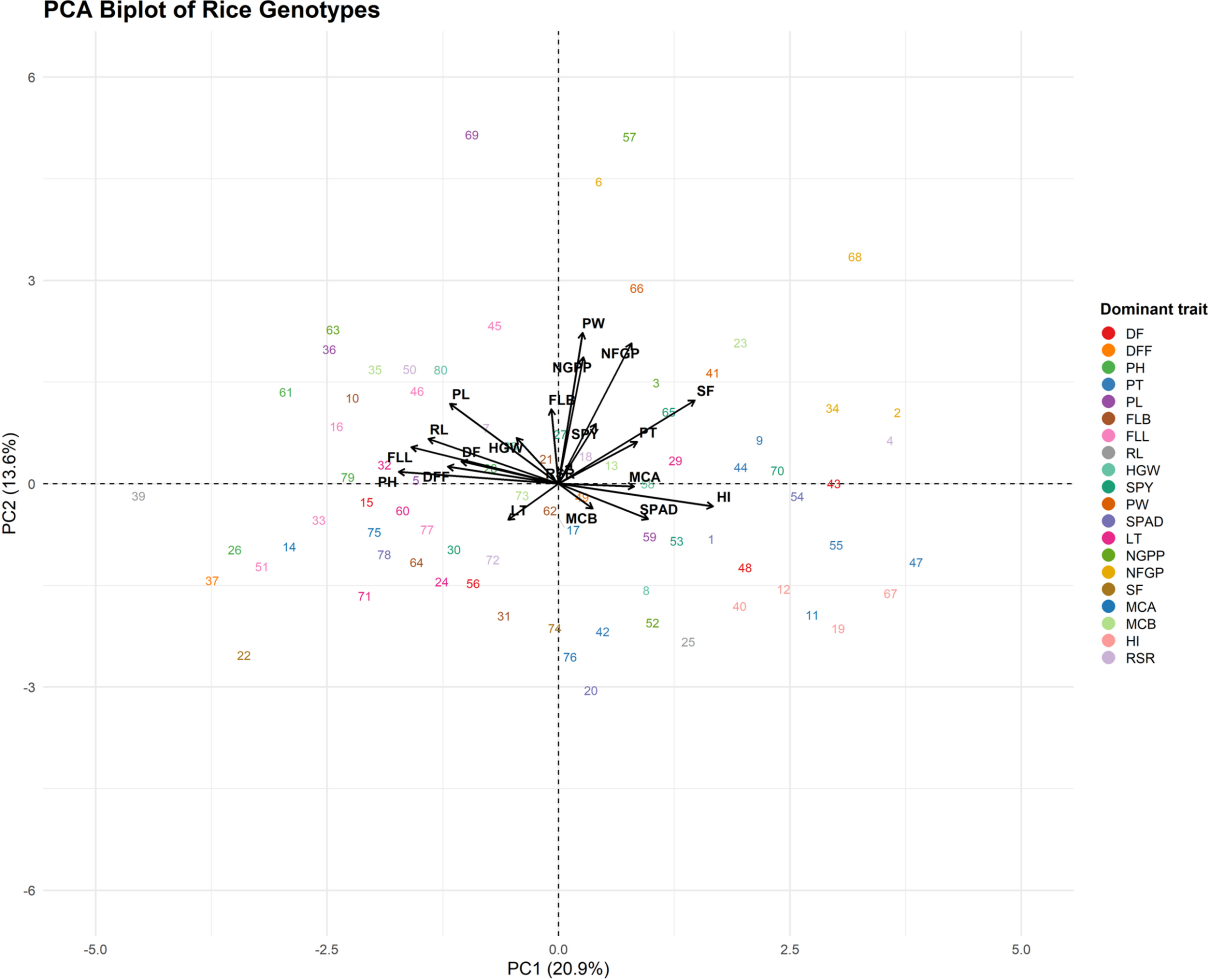
Principal component biplot of HTI-based trait values of the rice genotypes. PC1 and PC2 explained 20.9% and 13.6% of the total variation, respectively. The trait vectors indicate the direction and magnitude of contribution, while different colors of genotype labels denote their dominant associated traits.

The PCA biplot showed that the genotypes spread out throughout the four quadrants, which the PC1 and PC2 boundaries created, because the genotypes showed different genetic characteristics that reacted to heat stress (**Figure 4**). Genotypes positioned in the direction of traits grouped under the same color were strongly associated with those characters. Genotypes that occupied the PC1 positive area showed higher harvest index and spikelet fertility values, while the PC1 negative area contained genotypes that showed vegetative growth traits, including PH, FLL, RL, and PL. The positive PC2 axis was associated with panicle weight, number of grains per panicle, and number of filled grains per panicle, indicating their contribution to yield. The genotypes located on the negative PC2 axis showed reduced association with the reproductive components. The genotypes that were located close to the vectors of single plant yield, harvest index, spikelet fertility, and panicle weight showed better results in yield-related traits during heat stress conditions. The genotypes clustered around the leaf temperature were more strongly linked with vegetative growth traits than with yield-related traits.

### 3.7 Cluster analysis

**Figure 5:**
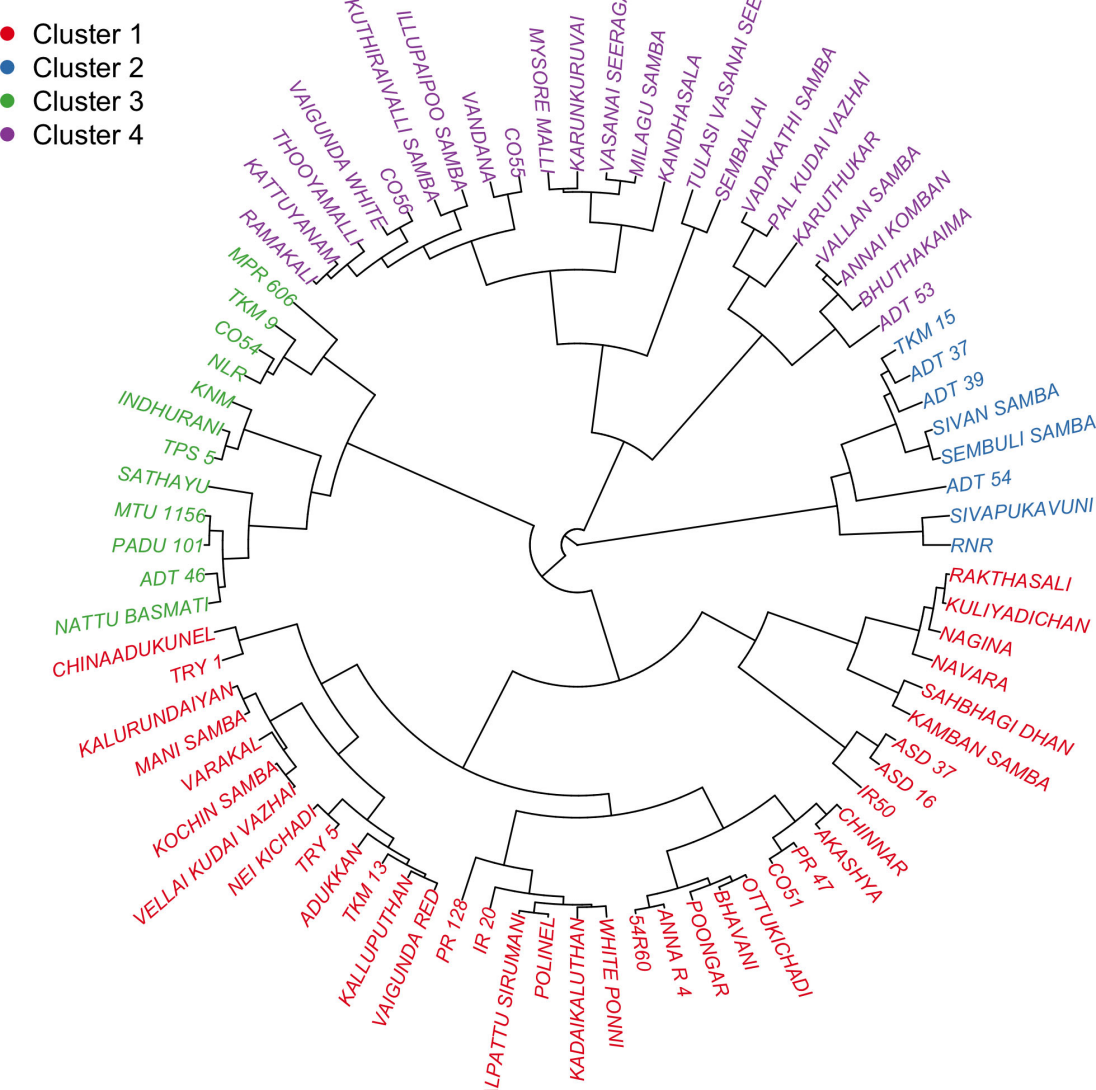
Circular dendrogram showing the clustering pattern of 80 rice genotypes based on selected HTI-related traits associated with heat tolerance and yield stability, generated using Mahalanobis D^2^ statistics.

The most significant yield-contributing and heat-responsive traits were further evaluated by cluster analysis based on the findings of principal component analysis, path coefficient analysis, and genotypic correlation. This was done to improve discriminatory efficiency among genotypes and decrease multicollinearity. The 80 rice genotypes were divided into four genetically distinct groups using these chosen traits (SPY, HI, NGPP, SF, and LT) (**Figure 5**; **Supplementary Table 2**). The Mahalanobis D^2^ cluster analysis showed significant variation in attributes relevant to yield and heat tolerance. There was significant genetic variety among the lines under evaluation, as seen by the unequal genotype distribution among clusters (**Table 8**). The biggest group (37 genotypes) was Cluster I, which had moderate mean values for SF (0.81), HI (1.08), NGPP (0.61), and SPY (0.59). The highest NGPP value of 2.01 was achieved by Cluster II, which included eight genotypes. The cluster showed a lower SPY value of 0.37 and a lower HI value of 0.48. The 12 genotypes of Cluster III demonstrated their best performance through an SPY value of 0.60 and a high HI value of 2.44, together with an above-average SF value of 0.84, which showed their capacity to distribute resources during heat stress conditions. The 23 genotypes of Cluster IV showed an SPY value of 0.37, an HI value of 0.47, and an SF value of 0.39, which demonstrated their weaker reproductive abilities. The intra-cluster distances varied from 5.95 in Cluster III to 7.74 in Cluster IV, demonstrating that Cluster III had the most homogeneous genotypes (**Supplementary Table 3**). The study established that clusters showed big genetic differences because their inter-cluster distances exceeded their intra-cluster distances. Clusters II and III exhibited the maximum inter-cluster divergence (13.55), while an identical value was noted between Clusters II and IV.

**Table 8:** Cluster mean values for selected yield-contributing and heat-responsive traits based on HTI values.

Cluster	1	2	3	4
SPY	0.59	0.37	0.60	0.37
HI	1.08	0.48	2.44	0.47
NGPP	0.61	2.01	1.10	0.77
SF	0.81	0.80	0.84	0.39
LT	1.14	1.17	1.11	1.18

For abbreviation check **Table 3**.

### 3.8 MGIDI analysis

The multi-trait genotype-ideotype distance index (MGIDI) was applied to simultaneously improve yield and heat tolerance-related traits under stress conditions. Since yield under heat stress is governed by multiple interrelated morphological, physiological, and reproductive traits, single-trait selection may lead to biased or unbalanced improvement. Therefore, MGIDI was used to combine all studied traits into a single selection index by reducing dimensionality through factor analysis and estimating the distance of each genotype from an ideal ideotype. In the MGIDI analysis, traits were assigned selection directions before ideotype construction, with most yield, reproductive, and growth-related traits considered desirable in the increasing direction, whereas DF, DFF, and LT were considered desirable in the decreasing direction under heat stress. Reduced MGIDI values reflected greater similarity of genotypes to the target ideotype because they combined favorable expression of traits selected for increase with reduced expression of traits selected for decrease. This approach enables balanced selection by minimizing the effect of multicollinearity among traits and identifying genotypes that combine desirable characteristics across multiple parameters.

Factor analysis extracted eight latent factors (FA1–FA8), with high communality values (0.56– 0.97), indicating that most of the variability in the characters was well explained by the factor structure. The results are presented in **Table 9**. FA1 grouped PH, FLL, RL, HI, PL, and SPAD, among which HI recorded a substantial positive selection gain of 21.52%, indicating improved assimilate partitioning efficiency. FA2 was dominated by grain number traits, particularly NGPP and NFGP, which exhibited very high positive selection gains of 58.01% and 85.63%, respectively, reflecting strong enhancement in reproductive sink capacity. FA3 was associated mainly with hundred-grain weight and panicle weight, with panicle weight exhibiting a notable gain of 59.09%. FA4 represented phenological traits DF and DFF, both of which recorded negative selection gains (−3.85% and −5.24%). Since lower values were defined as desirable, these reductions indicate relatively earlier flowering among selected genotypes under heat stress conditions. FA6 was strongly influenced by MCA (selection gain 24.22%), while FA7 grouped key yield traits, including SF, PT, and SPY. The SPY showed a positive gain of 22.35%, and SF improved by 22.27%, demonstrating coordinated improvement in reproductive stability and yield expression. Leaf temperature showed only minimal variation (1.05%), suggesting relative thermal stability among selected genotypes.

**Table 9:** Factor loadings, communality estimates, factor assignments and selection gains of HTI-based traits under MGIDI analysis.

Trait	FA1	FA2	FA3	FA4	FA5	FA6	FA7	FA8	Factor	SG %	Sense	Communality
DF	0.14	0.05	−0.01	0.97	−0.03	0.02	−0.06	0.01	FA4	−3.85	decrease	0.97
DFF	0.22	0.02	−0.02	0.94	−0.05	0.02	−0.08	0.08	FA4	−5.24	decrease	0.96
FLB	−0.05	−0.24	0.06	0.08	−0.04	−0.06	−0.02	0.84	FA8	35.59	increase	0.79
FLL	−0.59	0.14	−0.28	−0.20	0.05	−0.30	0.17	0.18	FA1	0.61	increase	0.64
HGW	−0.21	0.16	−0.84	0.01	−0.12	0.03	0.02	−0.06	FA3	11.02	increase	0.80
HI	0.74	0.12	−0.03	−0.04	0.12	0.37	−0.37	0.09	FA1	21.52	increase	0.85
LT	0.12	0.01	−0.42	−0.08	0.17	−0.02	−0.46	−0.35	FA7	1.05	decrease	0.56
MCA	0.12	−0.02	0.00	0.04	0.01	0.92	−0.06	−0.02	FA6	24.22	increase	0.87
MCB	0.13	0.06	−0.12	−0.05	−0.75	0.39	0.02	0.01	FA5	18.01	increase	0.76
NFGP	0.14	−0.94	−0.02	−0.03	0.00	−0.01	−0.20	0.08	FA2	85.63	increase	0.95
NGPP	0.00	−0.94	0.13	−0.09	0.05	0.02	0.08	0.16	FA2	58.01	increase	0.94
PH	−0.75	0.15	0.06	−0.25	−0.14	−0.18	0.08	0.08	FA1	−6.49	increase	0.71
PL	−0.61	−0.11	−0.26	−0.02	0.05	0.15	0.19	0.46	FA1	21.27	increase	0.72
PT	−0.02	−0.11	0.17	0.13	−0.08	0.06	−0.82	−0.04	FA7	13.97	increase	0.74
PW	−0.08	−0.59	−0.68	0.12	0.06	−0.01	−0.10	0.13	FA3	59.09	increase	0.87
RL	−0.79	0.05	−0.06	−0.08	0.08	0.10	−0.01	0.10	FA1	3.84	increase	0.67
RSR	0.04	−0.01	−0.05	−0.13	0.82	0.35	0.02	−0.05	FA5	−6.01	increase	0.82
SF	0.30	−0.38	−0.30	0.22	−0.12	0.13	−0.63	−0.13	FA7	22.27	increase	0.81
SPAD	0.63	−0.01	0.05	0.12	−0.14	0.02	0.22	0.30	FA1	2.33	increase	0.58
SPY	0.09	0.19	−0.39	−0.08	0.33	−0.05	−0.62	0.36	FA7	22.35	increase	0.83

For abbreviation check **Table 3**.

**Figure 6:**
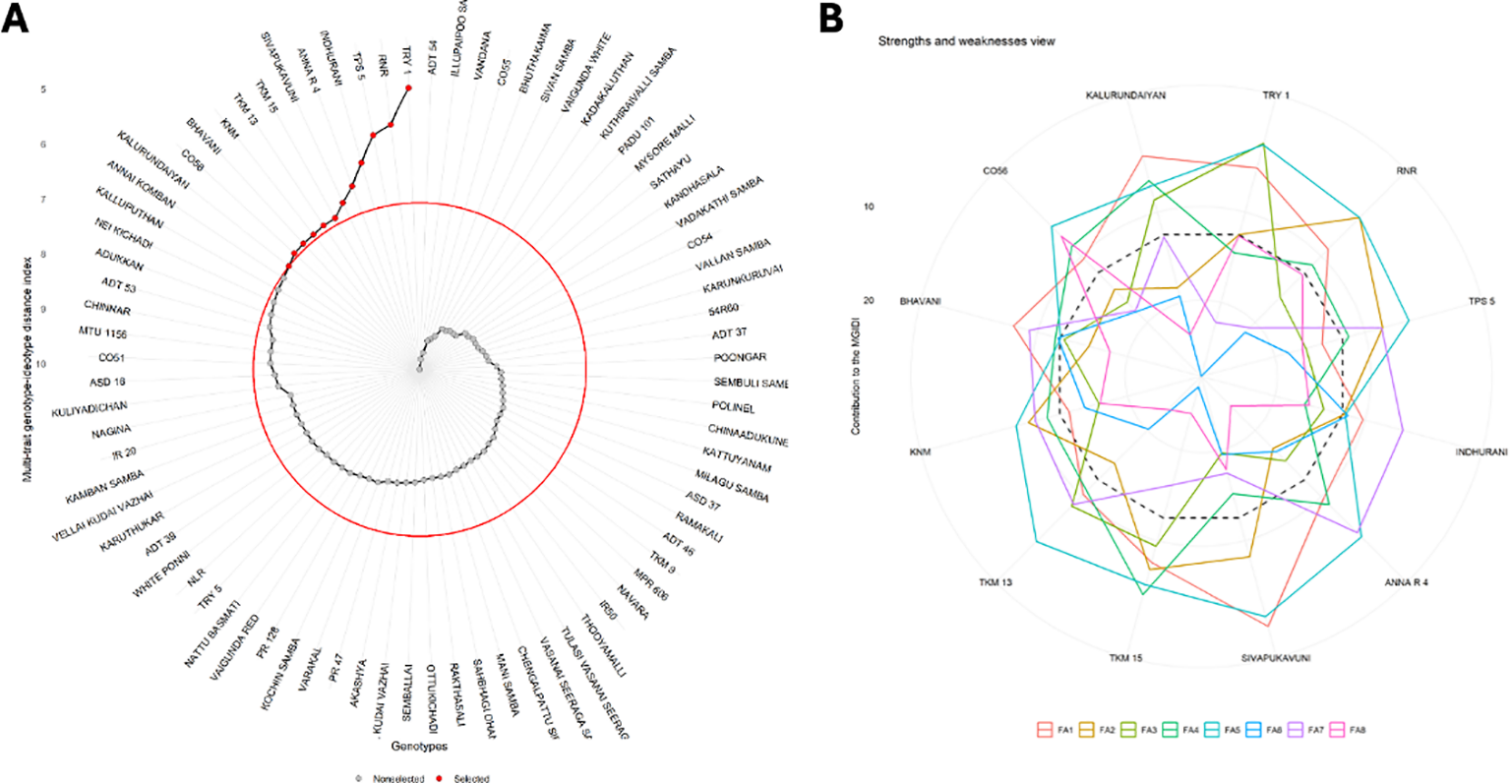
MGIDI-based genotype ranking and strength-weakness view of rice genotypes. **(A)** Circular plot showing MGIDI values and genotype rankings, with selected genotypes highlighted in red. **(B)** Strength-weakness view showing the contribution of latent factors (FA1-FA8) to the MGIDI values of selected genotypes.

The MGIDI ranking established different genotype categories based on their distance from the ideotype performance path, which showed that lower MGIDI values represented better multi-trait athletic capability. The genotype-wise ranking is presented in **Supplementary Table 4** and **Figure 6A**. The top-performing genotypes for this study included TRY 1 (4.98), RNR 15048 (5.62), TPS 5 (5.76), Indhurani (6.20), and Anna R 4 (6.55), which combined multiple factors to achieve high yield and grain production and effective reproductive processes and protection against heat stress damage. Based on MGIDI distance from the ideotype, ADT 54 (10.10), Illupaipoo Samba (9.92), Vandana (9.80), CO55 (9.56), and Bhuthakaima (9.52) presented their least appealing multi-trait excellence compared to other genotypes.

The strength-weakness view provided a graphical explanation of how eight latent factors contributed to the MGIDI values of the selected genotypes (**Figure 6B**). In general, the genotypes with smaller and more evenly distributed factor contributions were closer to the ideotype, whereas those with disproportionately high contributions from one or more factors reflected specific weaknesses in the corresponding trait groups. From the results, TRY 1, RNR 15048, TPS 5, Indhurani, and Anna R 4, which were ranked among the most desirable genotypes, showed relatively balanced contribution patterns, supporting their multi-trait superiority under heat stress.

### 3.9 Reproductive stage thermal exposure

Flowering window-based temperature summaries confirmed that genotypes under the stress environments experienced greater reproductive stage thermal load than those under the normal environments (**Supplementary Table 5**; **Supplementary Figure 1**). In general, Heat Stress Trial I showed a higher mean maximum temperature and a greater number of days with Tmax above 35°C and higher HDD values than Heat Stress Trial II, indicating stronger daytime heat stress, whereas Heat Stress Trial II also imposed clear reproductive stage heat stress but with a different thermal profile. These results indicate that the reduction in performance under stress was associated with actual reproductive stage heat exposure, although the magnitude of exposure varied among the genotypes. The group-wise summaries further showed that the early, intermediate, and late flowering genotypes all experienced substantial reproductive stage heat exposure under the stress environments. In Heat Stress Trial I, the average number of days with Tmax above 35°C was 12 days for early-flowering genotypes, 10 for intermediate-flowering genotypes, and 12 for late-flowering genotypes. In Heat Stress Trial II, early, intermediate, and late flowering genotypes experienced 8, 7, and 8 days with Tmax above 35°C, respectively (**Supplementary Table 6**; **Table 2**).

## 4 Discussion

High temperature during the reproductive stage remains a key limiting factor to stable rice productivity, and this study was undertaken to understand how diverse rice genotypes differ in their ability to maintain yield and related traits under stress. The pooled ANOVA revealed significant genotype, environment, and genotype × environment interaction effects for all traits, confirming substantial genetic variability and contrasting genotypic responses under heat stress. The non-significant replication and block effects showed that the alpha-lattice design efficiently controlled experimental variation, allowing reliable evaluation of genotypes (Redhu et al., 2025). Heat stress influenced all the studied traits, with the most severe reductions observed in reproductive and yield-related parameters such as SPY, PW, NFGP, SF, and PT. This confirms that the reproductive phase is highly sensitive to elevated temperature. The delayed DF and DFF suggest that increased temperature disrupted normal flowering behavior rather than promoting escape through early flowering. Previous studies also indicate that flowering responses under heat are not uniform, as high temperature may either accelerate or delay flowering depending on the species and stress (Chen et al., 2023; Tang et al., 2023). The increase in leaf temperature further indicates reduced transpirational cooling or impaired stomatal regulation, thereby increasing thermal load on reproductive tissues (Aryan et al., 2025; Hatfield and Prueger, 2015), while changes in moisture content and root-shoot ratio indicate that heat stress altered the internal growth balance of the plant, reflecting biomass accumulation and physiological adjustment under stress. In contrast, the relatively lower increase in SPAD suggests that chlorophyll degradation was not a key contributor to yield loss under heat stress in the evaluated panel. A parallel finding was also reported by Haque et al. (2021). Because the response to heat stress varied among genotypes, HTI was used to identify those genotypes that were able to sustain their performance under high temperature.

Higher HTI values indicate better relative performance under both normal and stress conditions, making the index useful for identifying genotypes with high yield and heat stress tolerance (Bhandari et al., 2024; Fernandez, 1992). The results of the present study revealed low HTI values for the yield-associated traits such as SPY, PW, PT, NFGP, and SF. This reflects the sensitivity of reproductive traits to high temperature (Chandramaniya et al., 2025; Lamba et al., 2023). On the other hand, wide variation in HTI values among genotypes indicated substantial genetic diversity for heat tolerance. The genotypes TKM 9, TRY 1, PR 128, TPS 5, and TRY 5 recorded higher HTI values for the target trait single plant yield, explaining better yield retention under stress.

Correlation analysis was computed using HTI values to explain the pattern of association among traits related to heat tolerance. The analysis revealed that SPY was positively correlated with PT, SF, and HI. This indicates the importance of reproductive efficiency for yield maintenance. The negative association between leaf temperature and yield further clarifies that higher thermal load was unfavorable for sustaining productivity. This is consistent with earlier reports that higher tissue temperature is correlated with greater heat damage, while cooler canopies or leaves support better performance under stress (Li et al., 2020). The strong positive correlation between the NGPP and further confirms the importance of grain filling in yield. In addition, spikelet fertility was positively correlated with the number of productive tillers, panicle weight and harvest index but negatively associated with leaf temperature and delayed flowering, indicating reduced reproductive stability under heat stress. This agrees with the findings of Aryan et al. (2025) that heat stress during the reproductive stage reduced spikelet fertility and adversely affected yield-related traits in rice. These findings suggest that spikelet fertility, panicle weight, harvest index, and number of productive tillers are useful selection traits for identifying genotypes that combine better heat tolerance with stable yield performance.

While correlation analysis revealed the association among HTI-based trait responses, path coefficient analysis helps in distinguishing the direct and indirect effects of individual traits on yield under heat stress (Khan et al., 2022). Among the traits studied, HI showed the strongest positive direct effect on SPY, explaining that better grain allocation was one of the major factors in sustaining yield under stress. This is important since heat stress often disrupts source-sink balance and reduces the efficient conversion of biomass into grain (Bag et al., 2026; Kobata et al., 2018). The NFGP and NGPP also displayed strong positive direct effects, additionally pointing out the importance of reproductive strength and grain filling in yield maintenance. This observation agrees with earlier reports that heat stress reduces the pollination process, spikelet fertility, and grain filling, which ultimately contribute to yield reduction (Lohiteswararao et al., 2021; Rehman et al., 2024). PW and PT also showed positive direct effects, signifying that genotypes can maintain panicle development and the number of productive tillers at the higher temperatures. In contrast, the DFF and LF showed a negative direct effect on yield, indicating that delayed flowering and thermal load were unfavorable under stress. This supports the observation that the stress-induced delays in reproductive timing may prolong the exposure of floral organs to high temperatures (Tang et al., 2023). Although spikelet fertility showed a positive effect on yield (0.337*), its direct contribution in the present path coefficient analysis was relatively low (0.004), suggesting that its effect on yield was closely associated with other yield-related traits such as HI, NFGP, PT and PW. Previous studies have shown that high-temperature stress substantially reduces spikelet fertility and harvest index, although the response varies among rice species, ecotypes, and cultivars (Prasad et al., 2006). Reduced spikelet fertility under heat stress has also been associated with impaired pollen production, abnormal anther development, and disrupted reproductive processes (Hu et al., 2021). Likewise, changes in spikelet fertility and grain traits under high temperature conditions have also been reported under alternate wetting and drying (AWD) management, emphasizing the importance of reproductive stability for maintaining rice yield under stress (Okpala et al., 2025). These findings suggest that spikelet fertility remained important for yield, but its effect was closely linked with other yield-related traits under the present study conditions. Likewise, plant height exhibited a relatively small positive effect on yield (0.054) but a strong negative effect through harvest index (-0.371). This suggests that increased vegetative growth did not necessarily translate into improved grain production under heat stress, likely due to reduced biomass partitioning efficiency toward grain formation, consistent with earlier reports under heat stress conditions (Raaj et al., 2018; Bhattarai et al., 2024). The relatively higher residual effect suggests that additional unmeasured factors may contribute to grain yield variation under heat stress. Traits such as pollen viability, spikelet tissue temperature, antioxidant defense activity, and membrane stability may also influence heat tolerance and represent important targets for future investigation.

Principal component analysis was executed to determine the contribution of the 20 HTI-based trait values to the total variation in heat tolerance response and to identify the major trait combinations connected with high yield. In the present study, eight principal components with eigenvalues more than 1 explained 79.17% of the total variability, indicating that the 20 studied traits effectively captured the major differences among genotypes. PC1 mainly separated vegetative and phenological traits such as PH, FLL, RL, and flowering time from reproductive efficiency traits such as harvest index and spikelet fertility. This implies that stronger vegetative growth and delayed flowering were not associated with better yield performance under stress. In addition to this, in a study on chickpea, the late flowering plants had higher biomass but low yield (Mallikarjuna et al., 2019). PC2 was mainly influenced by PW, NGPP, and NFGP, indicating that yield stability under stress depends on reproductive sink strength and grain-filling capacity. Thus, genotypes with favorable scores for components associated with reproductive efficiency are more desirable for selection under high temperature. The PCA biplot with PC1 and PC2 also supported this, as the genotypes located close to single plant yield, harvest index, spikelet fertility, and panicle weight appeared superior under heat stress, whereas those associated with leaf temperature and vegetative traits appeared less efficient in yield retention. These results emphasize reproductive efficiency as a key selection criterion under heat stress.

The cluster analysis was performed using selected key traits based on correlation, path coefficient analysis and PCA to improve genotype discrimination and reduce the effect of redundancy among the highly correlated variables. The HTI values of SPY, HI, NGPP, SF, and LT were used for cluster analysis. The accessions were separated into four clusters; similar cluster-based grouping was widely used in rice breeding to identify genetically distinct parents and to exploit heterotic gain through crossing of divergent groups (Rajan et al., 2023). In this study, Cluster III appeared to be the most promising group for breeding because it combined relatively higher single plant yield, harvest index and spikelet fertility, indicating better reproductive efficiency under stress. Cluster II, on the other hand, showed the highest number of grains per panicle, signifying sink potential, while its lower yield and harvest index indicated that this potential was not fully converted into stable productivity. The greater inter-cluster distances compared with intra-cluster distances further confirmed the breeding value of selecting parents from divergent clusters (Alawadi et al., 2025). In particular, the divergence between Cluster II and Cluster III suggests that crosses between these groups may generate recombinants combining higher grain number with better yield stability under heat stress.

Although correlation, path coefficient analysis, PCA, and cluster analysis provided important information on trait relationships, direct effects, and genetic divergence, they did not provide a single integrated basis for selecting genotypes with balanced superiority across all 20 traits. Therefore, MGIDI analysis was carried out to identify genotypes that combined high yield with heat tolerance by integrating all 20 studied traits into a single ideotype-based selection context. A related approach was also followed by Hasan et al. (2024). In the present study, factor analysis grouped the measured traits into eight latent factors, and the high communality values indicated that most of the variation was well explained by the structure. The positive selection gains observed for HI, NGPP, NFGP, PW, SF, and SPY indicate that the selected genotypes combined reproductive stability with agronomic productivity under heat stress. The top-ranked genotypes, TRY 1, RNR 15048, TPS 5, Indhurani, and Anna R 4, recorded lower MGIDI values, showing that they were closer to the ideal ideotype and possessed balanced superiority across multiple phenological, reproductive, and yield-associated traits. Consequently, the top-ranked genotypes may be considered promising donor parents or elite materials for improving heat tolerance and yield stability through multi-trait selection.

Comparison of the MGIDI-selected genotypes with the reference checks, Nagina 22 and Vandana, further clarified the relative strength of these selections under reproductive-stage heat stress. As expected, Vandana behaved as a susceptible check, recording a lower SPY-based HTI value of 0.28 and ranking among the poorly performing accessions in the integrated analysis with an MGIDI value of 9.80 (rank 78). Nagina 22, included as the tolerant check, performed better than Vandana, with an SPY-based HTI value of 0.51 and an MGIDI value of 7.71 (rank 23). Notably, however, several genotypes evaluated in the present study showed stronger performance under these field conditions. The genotypes TRY 1 and TPS 5 recorded higher SPY-based HTI values of 0.86 and 0.84, respectively, indicating better yield retention under stress than Nagina 22. In the integrated multi-trait analysis, TRY 1, TPS 5, RNR 15048, Indhurani, and Anna R 4 performed more favorably under stress conditions. Their superior performance appeared to be associated with favorable reproductive and yield-related trait combinations rather than a single contributing factor.

Although the selected genotypes showed overall superiority under heat stress, the underlying trait combinations differed among them. The genotype TPS 5 combined higher HTI values for PT (0.88), NGPP (1.75), NFGP (1.84), and HI (2.61), together with strong yield retention (SPY = 0.84). TRY 1 also showed high yield retention (SPY = 0.86), but its advantage appeared to be driven more by panicle productivity and grain filling, as reflected in higher HTI values for PW (1.19), NGPP (1.45), and NFGP (1.42), despite lower productive tillers. In addition, RNR 15048 showed only marginally higher SPY (0.52) than Nagina 22 (SPY = 0.51) but recorded high HTI values for PW (1.28), NGPP (2.76), and NFGP (2.33), which likely contributed to its favorable MGIDI rank. The landrace Indhurani appeared to show its superiority mainly through improved harvest index (2.78), together with high HTI values for NGPP and NFGP, whereas Anna R 4 was supported more by productive tillers (1.33) and harvest index (1.84). Therefore, these comparisons indicate that better performance under heat stress was achieved through different combinations of reproductive and yield-related traits across genotypes, rather than through a single common mechanism. Notably, the selected superior genotypes were distributed across different flowering groups, with TPS 5, Anna R 4, and Nagina 22 in the early flowering group and TRY 1, RNR 15048, and Indhurani in the intermediate flowering group, while Vandana belonged to the late flowering group. This suggests that performance under heat stress was not determined by flowering group alone, but through the ability of genotypes to sustain favorable reproductive and yield-related traits under stress.

The present results highlight the multifactorial nature of heat tolerance in rice phenological, reproductive, physiological, and yield-related traits. The combined results further indicate that reproductive stability, grain filling, and efficient assimilate partitioning are central to maintaining yield under stress. Accordingly, genotypes TRY 1, TPS 5, RNR 15048, Indhurani, and Anna R 4 may serve as promising materials for breeding programs aimed at improving yield stability under reproductive-stage heat stress.

## 5 Conclusion

Our research findings demonstrate that heat stress substantially reduced the performance of rice genotypes across multiple phenological, morphological, physiological, reproductive, and yield-related traits, with the most severe effects observed in reproductive stability and yield expression. SPY, PW, NFGP, SF, and PT emerged as the most heat-sensitive traits, while strong genotypic variation under stress highlighted the presence of useful diversity in breeding. Correlation and path coefficient analysis exhibited that harvest index, filled grains per panicle, panicle weight, spikelet fertility, and number of productive tillers were the major contributors to stable yield under heat stress, whereas delayed flowering and higher leaf temperature were associated with poor performance. MGIDI further identified genotypes such as TRY 1, TPS 5, RNR 15048, Indhurani, and Anna R 4 as promising materials with balanced superiority across multiple traits. Therefore, this study shows that a multi-trait approach is essential for identifying high-yielding and heat-tolerant rice genotypes and that the integration of HTI with multivariate analyses provides a valuable framework for breeding climate-resilient rice. Further evaluation is needed to confirm the consistency and adaptability of the selected genotypes across locations.

## Data Availability

The original contributions presented in the study are included in the article/**Supplementary Material**. Further inquiries can be directed to the corresponding author.
