# Supplementary material for: Identification of rice genotypes for reproductive stage heat tolerance and yield stability through multi-trait and multivariate analysis
Source: Front Plant Sci. 2026 Jun 17;17:1853773. doi: 10.3389/fpls.2026.1853773 (PMC13318890; doi:10.3389/fpls.2026.1853773)
Supplement: Supplementary file 2 [file Table2.docx]

Supplementary Tables

**Supplementary Table. 1** HTI based mean performance of rice genotypes for 20 phenological, morphological, physiological, reproductive and yield related traits

| **Genotypes** | **DF** | **DFF** | **PH** | **PT** | **PL** | **FLB** | **FLL** | **RL** | **HGW** | **SPY** | **PW** | **SPAD** | **LT** | **NGPP** | **NFGP** | **SF** | **MCA** | **MCB** | **HI** | **RSR** |
| --- | --- | --- | --- | --- | --- | --- | --- | --- | --- | --- | --- | --- | --- | --- | --- | --- | --- | --- | --- | --- |
| 54R60 | 1.18 | 1.12 | 0.58 | 0.31 | 0.91 | 1.08 | 0.76 | 0.45 | 0.59 | 0.57 | 0.37 | 1.41 | 1.12 | 0.71 | 0.63 | 0.91 | 0.89 | 0.60 | 1.53 | 0.55 |
| ADT 37 | 0.92 | 0.92 | 0.71 | 0.84 | 0.49 | 0.78 | 0.35 | 0.47 | 0.34 | 0.35 | 0.74 | 1.25 | 1.17 | 2.05 | 2.06 | 1.03 | 0.59 | 2.64 | 0.78 | 0.45 |
| ADT 39 | 1.16 | 1.14 | 0.55 | 0.85 | 0.82 | 1.39 | 0.75 | 0.93 | 0.17 | 0.23 | 0.37 | 0.99 | 1.09 | 1.90 | 1.45 | 0.78 | 1.38 | 1.47 | 0.25 | 0.30 |
| ADT 46 | 1.22 | 1.16 | 0.54 | 1.09 | 0.51 | 0.76 | 0.40 | 0.58 | 0.48 | 0.55 | 0.52 | 1.17 | 1.09 | 1.18 | 1.11 | 0.98 | 1.22 | 0.43 | 2.49 | 5.21 |
| ADT 53 | 1.02 | 0.99 | 1.03 | 0.28 | 1.36 | 0.58 | 1.48 | 1.36 | 0.76 | 0.73 | 0.52 | 1.04 | 1.11 | 0.74 | 0.22 | 0.30 | 1.58 | 0.93 | 1.33 | 2.94 |
| ADT 54 | 1.49 | 1.41 | 0.97 | 0.80 | 0.90 | 0.91 | 0.76 | 0.63 | 0.16 | 0.29 | 0.61 | 1.03 | 1.11 | 3.07 | 3.08 | 1.03 | 0.26 | 0.20 | 0.16 | 0.29 |
| ADUKKAN | 1.24 | 1.23 | 0.97 | 0.48 | 1.03 | 0.85 | 0.86 | 1.23 | 1.17 | 0.77 | 0.52 | 0.92 | 1.03 | 0.56 | 0.40 | 0.75 | 1.31 | 1.77 | 1.21 | 2.95 |
| AKASHYA | 0.95 | 0.91 | 0.75 | 0.70 | 0.79 | 0.89 | 0.65 | 1.00 | 0.12 | 0.50 | 0.08 | 0.95 | 1.13 | 0.76 | 0.48 | 0.64 | 1.52 | 1.23 | 1.01 | 0.97 |
| ANNA R 4 | 1.08 | 1.08 | 0.67 | 1.33 | 1.01 | 0.95 | 0.60 | 0.74 | 1.13 | 0.65 | 0.46 | 1.03 | 1.13 | 0.72 | 0.73 | 1.03 | 2.06 | 0.54 | 1.84 | 0.54 |
| ANNAI KOMBAN | 1.04 | 1.10 | 1.53 | 0.79 | 1.09 | 1.65 | 1.35 | 1.11 | 0.79 | 0.72 | 0.61 | 0.93 | 1.15 | 0.72 | 0.28 | 0.40 | 0.98 | 0.42 | 0.26 | 1.24 |
| ASD 16 | 0.82 | 0.83 | 0.61 | 0.76 | 1.06 | 0.79 | 0.63 | 0.65 | 0.72 | 0.21 | 0.27 | 1.20 | 1.22 | 0.38 | 0.32 | 0.88 | 2.39 | 0.79 | 1.85 | 1.02 |
| ASD 37 | 1.18 | 1.08 | 0.60 | 1.01 | 0.62 | 1.03 | 0.48 | 0.51 | 0.47 | 0.34 | 0.22 | 1.07 | 1.16 | 0.41 | 0.35 | 0.89 | 1.35 | 1.08 | 2.59 | 0.70 |
| BHAVANI | 1.18 | 1.15 | 1.18 | 0.89 | 1.07 | 1.11 | 0.81 | 0.67 | 0.82 | 0.50 | 0.47 | 1.02 | 1.18 | 0.59 | 0.57 | 0.96 | 1.73 | 2.82 | 1.32 | 0.84 |
| BHUTHAKAIMA | 1.29 | 1.29 | 1.33 | 0.48 | 0.98 | 0.78 | 1.44 | 0.84 | 0.15 | 0.69 | 0.06 | 0.88 | 1.08 | 0.70 | 0.33 | 0.47 | 0.36 | 0.46 | 0.11 | 0.96 |
| CHENGALPATTU SIRUMANI | 1.64 | 1.58 | 0.92 | 0.99 | 1.13 | 1.38 | 0.75 | 1.15 | 0.63 | 0.62 | 0.21 | 0.90 | 1.22 | 0.44 | 0.24 | 0.56 | 1.50 | 2.11 | 1.12 | 0.85 |
| CHINAADUKUNEL | 1.32 | 1.24 | 1.44 | 1.24 | 0.90 | 0.99 | 1.68 | 1.27 | 0.59 | 0.82 | 0.27 | 0.99 | 1.23 | 0.86 | 0.57 | 0.66 | 0.27 | 1.28 | 0.39 | 0.35 |
| CHINNAR | 1.12 | 1.06 | 0.91 | 0.21 | 1.03 | 0.83 | 1.38 | 0.70 | 0.87 | 0.49 | 0.46 | 0.95 | 1.08 | 0.65 | 0.43 | 0.67 | 2.34 | 1.98 | 1.45 | 0.43 |
| CO51 | 1.22 | 1.21 | 0.74 | 0.88 | 1.04 | 1.01 | 1.07 | 0.61 | 1.00 | 0.67 | 0.39 | 0.97 | 1.13 | 0.54 | 0.40 | 0.74 | 1.44 | 0.99 | 1.67 | 5.33 |
| CO54 | 1.06 | 1.07 | 0.45 | 0.48 | 0.57 | 0.73 | 0.57 | 0.50 | 0.57 | 0.66 | 0.31 | 1.07 | 1.09 | 0.39 | 0.32 | 0.84 | 0.80 | 2.19 | 3.14 | 0.17 |
| CO55 | 1.15 | 1.10 | 0.80 | 0.47 | 0.66 | 0.86 | 0.59 | 0.56 | 0.66 | 0.40 | 0.17 | 1.27 | 1.31 | 0.53 | 0.16 | 0.32 | 0.77 | 0.92 | 1.08 | 0.31 |
| CO56 | 1.07 | 1.09 | 0.72 | 0.79 | 1.45 | 1.89 | 0.84 | 0.47 | 0.72 | 0.33 | 0.37 | 1.16 | 1.22 | 1.19 | 0.41 | 0.38 | 1.35 | 1.48 | 0.88 | 1.04 |
| ILLUPAIPOO SAMBA | 1.38 | 1.44 | 1.42 | 0.26 | 0.67 | 0.91 | 0.98 | 1.17 | 1.24 | 0.15 | 0.10 | 1.18 | 1.17 | 0.59 | 0.10 | 0.12 | 0.68 | 2.46 | 0.13 | 0.53 |
| INDHURANI | 1.22 | 1.16 | 0.99 | 0.73 | 1.04 | 1.19 | 0.66 | 1.16 | 0.49 | 0.77 | 0.61 | 1.02 | 1.12 | 1.34 | 1.39 | 1.04 | 1.67 | 3.06 | 2.78 | 0.77 |
| IR 20 | 1.13 | 1.12 | 0.73 | 0.29 | 1.21 | 1.08 | 0.97 | 1.13 | 0.30 | 0.52 | 0.14 | 1.04 | 1.31 | 0.41 | 0.23 | 0.59 | 1.40 | 0.81 | 0.81 | 2.09 |
| IR 50 | 0.83 | 0.83 | 0.76 | 0.66 | 0.48 | 0.51 | 0.37 | 1.58 | 0.89 | 0.27 | 0.25 | 1.09 | 1.29 | 0.38 | 0.33 | 0.88 | 1.78 | 1.44 | 1.28 | 1.04 |
| KADAIKALUTHAN | 1.32 | 1.29 | 1.64 | 0.40 | 0.76 | 0.73 | 1.55 | 1.24 | 0.25 | 0.58 | 0.22 | 0.92 | 1.19 | 0.35 | 0.20 | 0.57 | 0.70 | 0.62 | 0.30 | 0.40 |
| KALLUPUTHAN | 1.29 | 1.28 | 0.99 | 0.65 | 0.94 | 0.82 | 0.81 | 0.98 | 1.03 | 0.86 | 0.51 | 1.01 | 1.11 | 0.60 | 0.49 | 0.83 | 1.74 | 1.13 | 1.50 | 2.86 |
| KALURUNDAIYAN | 1.00 | 1.02 | 1.54 | 0.71 | 1.11 | 0.85 | 0.80 | 1.02 | 1.33 | 0.58 | 0.64 | 1.04 | 1.11 | 0.55 | 0.39 | 0.75 | 1.31 | 1.65 | 0.61 | 0.92 |
| KAMBAN SAMBA | 1.21 | 1.14 | 0.90 | 0.45 | 0.77 | 0.90 | 0.80 | 0.79 | 0.75 | 0.44 | 0.43 | 0.95 | 0.99 | 0.87 | 0.78 | 0.93 | 1.75 | 1.49 | 1.70 | 1.39 |
| KANDHASALA | 1.19 | 1.11 | 0.67 | 0.06 | 1.11 | 0.69 | 1.23 | 0.53 | 0.42 | 0.04 | 0.45 | 1.06 | 1.29 | 1.23 | 0.63 | 0.53 | 1.43 | 1.97 | 0.04 | 0.39 |
| KARUNKURUVAI | 1.33 | 1.30 | 1.34 | 0.87 | 0.95 | 0.43 | 1.00 | 0.78 | 0.67 | 0.36 | 0.22 | 1.21 | 1.13 | 0.23 | 0.15 | 0.69 | 1.38 | 1.11 | 1.11 | 0.36 |
| KARUTHUKAR | 1.26 | 1.23 | 1.32 | 0.85 | 0.90 | 1.19 | 0.86 | 1.41 | 0.86 | 0.51 | 0.43 | 1.00 | 0.93 | 0.61 | 0.24 | 0.40 | 1.09 | 1.76 | 0.29 | 1.05 |
| KATTUYANAM | 1.10 | 1.06 | 1.53 | 0.54 | 1.00 | 0.80 | 1.70 | 0.88 | 0.93 | 0.36 | 0.28 | 0.93 | 1.15 | 0.80 | 0.33 | 0.42 | 0.77 | 1.69 | 0.17 | 0.60 |
| KNM 1638 | 1.13 | 1.08 | 0.57 | 0.90 | 0.76 | 1.16 | 0.68 | 0.62 | 0.34 | 0.60 | 0.53 | 1.16 | 1.17 | 1.56 | 1.37 | 0.89 | 1.78 | 2.13 | 1.65 | 0.89 |
| KOCHIN SAMBA | 1.17 | 1.17 | 1.53 | 0.89 | 1.16 | 0.95 | 1.01 | 1.18 | 0.81 | 0.53 | 0.80 | 0.96 | 1.13 | 0.92 | 0.60 | 0.66 | 0.54 | 0.15 | 0.13 | 0.50 |
| KULIYADICHAN | 1.35 | 1.41 | 1.49 | 1.13 | 1.59 | 0.28 | 1.10 | 1.54 | 1.33 | 0.38 | 0.71 | 1.02 | 1.08 | 0.64 | 0.65 | 1.02 | 1.56 | 1.78 | 0.37 | 0.67 |
| KUTHIRAIVALLI SAMBA | 1.53 | 1.53 | 1.20 | 0.39 | 0.80 | 0.94 | 1.62 | 1.05 | 0.79 | 0.39 | 0.41 | 1.09 | 1.19 | 0.29 | 0.05 | 0.19 | 1.19 | 1.18 | 0.38 | 2.11 |
| MANI SAMBA | 1.14 | 1.16 | 1.19 | 0.70 | 0.98 | 0.57 | 1.01 | 0.94 | 1.76 | 0.69 | 0.68 | 0.99 | 1.07 | 0.47 | 0.41 | 0.89 | 0.93 | 1.38 | 0.72 | 0.47 |
| MILAGU SAMBA | 1.38 | 1.39 | 1.65 | 0.40 | 1.20 | 0.80 | 1.16 | 1.56 | 1.24 | 0.22 | 0.24 | 0.86 | 1.15 | 0.61 | 0.30 | 0.53 | 1.10 | 0.57 | 0.06 | 1.33 |
| MPR 606 | 1.17 | 1.20 | 0.73 | 0.80 | 0.73 | 1.06 | 0.55 | 0.54 | 0.26 | 0.52 | 0.22 | 1.09 | 1.23 | 0.68 | 0.44 | 0.67 | 1.32 | 1.40 | 3.35 | 1.06 |
| MTU 1156 | 1.21 | 1.17 | 0.56 | 0.60 | 0.87 | 1.06 | 0.70 | 0.65 | 0.88 | 0.46 | 1.20 | 1.16 | 1.07 | 1.35 | 0.98 | 0.74 | 1.50 | 1.09 | 1.73 | 1.71 |
| MYSORE MALLI | 1.04 | 1.03 | 0.44 | 0.10 | 0.91 | 0.69 | 1.17 | 0.49 | 0.39 | 0.12 | 0.24 | 1.12 | 1.13 | 0.48 | 0.31 | 0.66 | 1.43 | 2.03 | 0.30 | 0.37 |
| NAGINA 22 | 0.63 | 0.65 | 0.97 | 0.69 | 0.50 | 1.03 | 0.45 | 0.85 | 1.00 | 0.51 | 0.79 | 1.04 | 1.11 | 0.71 | 0.79 | 1.13 | 0.95 | 1.99 | 1.19 | 0.69 |
| NATTU BASMATI | 1.09 | 1.08 | 0.60 | 1.24 | 0.67 | 0.67 | 0.51 | 0.85 | 0.70 | 0.47 | 0.59 | 0.89 | 1.11 | 1.01 | 0.87 | 0.88 | 1.63 | 0.87 | 1.55 | 0.45 |
| NAVARA | 1.03 | 0.99 | 0.69 | 0.81 | 0.94 | 0.74 | 1.80 | 1.01 | 1.17 | 0.61 | 1.21 | 0.79 | 1.08 | 0.48 | 0.51 | 1.08 | 0.44 | 0.82 | 0.29 | 0.54 |
| NEI KICHADI | 1.18 | 1.20 | 1.23 | 0.93 | 1.00 | 1.20 | 1.41 | 1.16 | 1.22 | 0.79 | 0.57 | 0.89 | 1.12 | 0.66 | 0.56 | 0.87 | 0.78 | 2.00 | 0.78 | 0.71 |
| NLR 3238 | 1.08 | 1.03 | 0.46 | 1.38 | 0.61 | 0.92 | 0.39 | 0.64 | 0.27 | 0.65 | 0.19 | 1.18 | 1.04 | 0.91 | 0.65 | 0.74 | 1.84 | 1.38 | 2.88 | 1.21 |
| OTTUKICHADI | 0.79 | 0.86 | 0.87 | 1.21 | 0.85 | 0.67 | 0.78 | 0.59 | 0.70 | 0.49 | 0.45 | 1.22 | 1.17 | 0.45 | 0.39 | 0.88 | 0.84 | 1.48 | 0.97 | 1.28 |
| PADU 101 | 1.42 | 1.41 | 0.64 | 0.41 | 0.74 | 0.60 | 0.77 | 0.72 | 0.41 | 0.35 | 0.30 | 0.98 | 1.08 | 1.32 | 1.05 | 0.81 | 1.22 | 0.72 | 1.57 | 1.95 |
| PAL KUDAI VAZHAI | 1.43 | 1.38 | 1.05 | 0.50 | 1.05 | 0.59 | 0.97 | 1.06 | 1.35 | 0.39 | 1.07 | 1.01 | 1.09 | 1.45 | 0.68 | 0.46 | 1.78 | 1.03 | 0.77 | 4.00 |
| POLINEL | 1.43 | 1.36 | 1.11 | 0.45 | 0.93 | 0.74 | 1.58 | 1.25 | 0.56 | 0.62 | 0.11 | 0.84 | 1.22 | 0.46 | 0.23 | 0.50 | 1.04 | 2.28 | 0.68 | 0.89 |
| POONGAR | 1.26 | 1.18 | 0.87 | 0.75 | 0.70 | 0.69 | 0.56 | 0.58 | 0.74 | 0.51 | 0.22 | 1.05 | 1.16 | 0.20 | 0.18 | 0.88 | 1.28 | 1.77 | 1.64 | 0.62 |
| PR 128 | 1.19 | 1.12 | 0.54 | 0.50 | 0.68 | 0.92 | 0.58 | 0.63 | 0.71 | 0.85 | 0.57 | 0.90 | 1.22 | 0.51 | 0.38 | 0.74 | 1.15 | 1.74 | 2.11 | 0.76 |
| PR 47 | 1.06 | 1.03 | 0.48 | 0.52 | 0.70 | 1.18 | 0.63 | 0.67 | 0.76 | 0.73 | 0.55 | 1.21 | 1.08 | 0.59 | 0.49 | 0.84 | 0.79 | 1.45 | 2.23 | 2.22 |
| RAKTHASALI | 0.96 | 0.94 | 0.56 | 1.83 | 0.68 | 0.81 | 0.49 | 0.74 | 0.31 | 0.40 | 0.29 | 0.97 | 1.09 | 0.49 | 0.51 | 1.06 | 1.68 | 2.16 | 0.82 | 1.14 |
| RAMAKALI | 0.94 | 0.97 | 0.72 | 0.42 | 0.92 | 0.88 | 0.75 | 1.24 | 0.35 | 0.38 | 0.13 | 0.92 | 1.19 | 0.59 | 0.24 | 0.42 | 1.22 | 0.68 | 0.20 | 2.28 |
| RNR 15048 | 1.13 | 1.06 | 0.79 | 0.58 | 1.11 | 1.81 | 0.93 | 1.19 | 0.65 | 0.52 | 1.28 | 1.10 | 1.26 | 2.76 | 2.33 | 0.87 | 1.54 | 1.40 | 0.87 | 1.40 |
| SAHBHAGI DHAN | 1.12 | 1.11 | 0.72 | 0.67 | 0.94 | 0.88 | 0.80 | 0.46 | 1.76 | 0.49 | 0.60 | 1.04 | 1.00 | 0.57 | 0.49 | 0.88 | 1.46 | 2.59 | 0.73 | 0.18 |
| SATHAYU | 1.42 | 1.40 | 0.82 | 0.40 | 0.44 | 0.69 | 0.85 | 0.65 | 0.64 | 0.33 | 0.48 | 1.05 | 1.07 | 1.21 | 0.66 | 0.56 | 1.99 | 2.12 | 2.18 | 0.97 |
| SEMBALLAI | 1.26 | 1.22 | 1.28 | 0.53 | 0.99 | 1.18 | 0.85 | 1.08 | 0.32 | 0.22 | 0.49 | 1.13 | 1.45 | 1.37 | 0.56 | 0.39 | 1.50 | 1.87 | 0.17 | 0.67 |
| SEMBULI SAMBA | 1.24 | 1.38 | 1.71 | 0.88 | 1.14 | 0.98 | 1.34 | 0.96 | 0.44 | 0.32 | 0.55 | 0.87 | 1.17 | 1.29 | 0.74 | 0.59 | 0.47 | 0.50 | 0.08 | 0.90 |
| SIVAN SAMBA | 1.34 | 1.52 | 0.89 | 0.70 | 0.59 | 0.26 | 0.58 | 0.86 | 0.49 | 0.32 | 0.34 | 0.79 | 1.17 | 1.45 | 0.94 | 0.68 | 1.79 | 1.29 | 0.78 | 3.34 |
| SIVAPUKAVUNI | 1.29 | 1.27 | 1.38 | 0.63 | 1.21 | 1.34 | 1.10 | 1.19 | 0.65 | 0.47 | 0.72 | 0.91 | 1.28 | 2.00 | 1.03 | 0.52 | 1.41 | 0.91 | 0.37 | 1.50 |
| THOOYAMALLI | 1.18 | 1.22 | 1.37 | 0.67 | 1.10 | 0.38 | 0.93 | 1.05 | 0.43 | 0.48 | 0.29 | 0.99 | 1.21 | 0.91 | 0.41 | 0.44 | 1.36 | 1.89 | 0.33 | 1.69 |
| TKM 13 | 1.09 | 1.09 | 0.62 | 0.89 | 0.74 | 0.85 | 1.14 | 0.78 | 1.01 | 0.86 | 0.75 | 1.00 | 1.08 | 1.05 | 0.81 | 0.79 | 1.57 | 1.68 | 1.14 | 1.33 |
| TKM 15 | 0.95 | 0.92 | 0.71 | 0.58 | 0.99 | 1.17 | 1.17 | 0.99 | 0.82 | 0.47 | 1.06 | 1.00 | 1.11 | 1.57 | 1.39 | 0.90 | 0.94 | 1.42 | 0.58 | 0.61 |
| TKM 9 | 1.09 | 1.05 | 0.36 | 0.71 | 0.54 | 0.59 | 0.79 | 0.51 | 0.58 | 0.93 | 0.27 | 1.23 | 1.08 | 0.52 | 0.44 | 0.86 | 1.58 | 1.23 | 3.35 | 2.24 |
| TPS 5 | 1.08 | 1.04 | 0.63 | 0.88 | 1.01 | 1.22 | 0.91 | 0.90 | 0.36 | 0.84 | 0.80 | 1.15 | 1.11 | 1.75 | 1.84 | 1.08 | 1.83 | 0.73 | 2.61 | 2.44 |
| TRY 1 | 1.16 | 1.09 | 0.75 | 0.48 | 1.79 | 1.55 | 1.39 | 1.32 | 1.28 | 0.86 | 1.19 | 1.04 | 1.16 | 1.45 | 1.42 | 0.97 | 1.24 | 1.06 | 0.65 | 2.04 |
| TRY 5 | 1.12 | 1.12 | 0.51 | 1.01 | 0.64 | 0.72 | 0.71 | 0.51 | 0.77 | 0.83 | 0.60 | 0.87 | 1.11 | 0.62 | 0.63 | 1.04 | 1.45 | 0.99 | 1.40 | 1.36 |
| TULASI VASANAI SEERAGA SAMBA | 1.03 | 1.06 | 1.43 | 0.30 | 1.21 | 1.37 | 0.90 | 0.97 | 0.42 | 0.18 | 0.12 | 1.16 | 1.36 | 1.51 | 0.23 | 0.15 | 1.33 | 2.24 | 0.42 | 0.53 |
| VADAKATHI SAMBA | 1.08 | 1.10 | 1.03 | 0.55 | 0.92 | 0.81 | 0.69 | 0.88 | 0.11 | 0.46 | 0.08 | 0.86 | 1.10 | 1.03 | 0.26 | 0.26 | 1.23 | 0.20 | 0.60 | 4.36 |
| VAIGUNDA RED | 1.23 | 1.22 | 1.32 | 0.65 | 0.85 | 1.07 | 0.84 | 0.84 | 0.35 | 0.69 | 0.38 | 1.08 | 1.13 | 0.79 | 0.55 | 0.71 | 1.29 | 0.48 | 1.00 | 2.04 |
| VAIGUNDA WHITE | 0.97 | 0.98 | 0.46 | 0.64 | 0.60 | 0.93 | 0.84 | 0.84 | 0.40 | 0.32 | 0.20 | 0.90 | 1.16 | 0.74 | 0.24 | 0.34 | 0.62 | 0.72 | 0.77 | 0.75 |
| VALLAN SAMBA | 1.23 | 1.20 | 0.95 | 0.45 | 0.74 | 0.93 | 0.83 | 1.17 | 1.19 | 0.63 | 0.46 | 1.07 | 1.16 | 0.44 | 0.14 | 0.32 | 0.43 | 0.50 | 0.29 | 0.91 |
| VANDANA | 1.19 | 1.15 | 0.56 | 0.49 | 0.76 | 0.79 | 0.64 | 0.54 | 0.66 | 0.28 | 0.16 | 1.14 | 1.23 | 0.40 | 0.15 | 0.39 | 0.37 | 1.05 | 1.00 | 1.16 |
| VARAKAL | 1.11 | 1.09 | 1.28 | 0.66 | 0.81 | 1.03 | 1.36 | 1.20 | 0.87 | 0.55 | 0.25 | 1.11 | 1.08 | 0.31 | 0.17 | 0.55 | 1.27 | 1.14 | 0.34 | 0.95 |
| VASANAI SEERAGA SAMBA | 1.03 | 1.02 | 1.36 | 0.45 | 1.15 | 0.92 | 0.87 | 0.78 | 0.90 | 0.24 | 0.42 | 0.84 | 1.21 | 0.56 | 0.25 | 0.45 | 1.09 | 2.19 | 0.12 | 0.33 |
| VELLAI KUDAI VAZHAI | 1.11 | 1.09 | 1.65 | 0.76 | 1.30 | 1.28 | 1.16 | 0.94 | 0.57 | 0.51 | 0.22 | 1.04 | 1.14 | 0.79 | 0.46 | 0.57 | 0.82 | 0.38 | 0.12 | 1.15 |
| WHITE PONNI | 1.15 | 1.25 | 0.63 | 0.73 | 1.17 | 0.94 | 0.89 | 0.76 | 1.81 | 0.57 | 1.15 | 0.89 | 1.25 | 0.77 | 0.50 | 0.67 | 0.60 | 0.48 | 0.45 | 1.07 |

**Supplementary Table. 2** Inter and intra cluster distances among rice genotype clusters based on Mahalanobis D^2^ analysis

| **Cluster** | **1** | **2** | **3** | **4** |
| --- | --- | --- | --- | --- |
| **1** | **6.41** | 13.12 | 10.75 | 10.26 |
| **2** | 13.12 | **6.30** | 13.55 | 13.55 |
| **3** | 10.75 | 13.55 | **5.95** | 13.12 |
| **4** | 10.26 | 13.55 | 13.12 | **7.74** |

**Supplementary Table. 3** Distribution of rice genotypes into different clusters based on selected HTI related traits

| **Cluster** | **No of Genotypes** | **Genotypes** |
| --- | --- | --- |
| **I** | 37 | 54R60, Adukkan, Akashya, Anna R 4, ASD 16, ASD 37, Bhavani, Chengalpattu Sirumani, Chinaadukunel, Chinnar, CO51, IR 20, IR50, Kadaikaluthan, Kalluputhan, Kalurundaiyan, Kamban Samba, Kochin Samba, Kuliyadichan, Mani Samba, Nagina 22, Navara, Nei Kichadi, Ottukichadi, Polinel, Poongar, PR 128, PR 47, Rakthasali, Sahbhagi Dhan, TKM 13, TRY 1, TRY 5, Vaigunda Red, Varakal, Vellai Kudai Vazhai, White Ponni |
| **II** | 8 | ADT 37, ADT 39, ADT 54, RNR 15048, Sembuli Samba, Sivan Samba, Sivapukavuni, TKM 15. |
| **III** | 12 | ADT 46, CO54, Indhurani, KNM 1638, MPR 606, MTU 1156, Nattu Basmati, NLR 3238, Padu 101, Sathayu, TKM 9, TPS 5. |
| **IV** | 23 | ADT 53, Annai Komban, Bhuthakaima, CO55, CO56, Illupaipoo Samba, Kandhasala, Karunkuruvai, Karuthukar, Kattuyanam, Kuthiraivalli Samba, Milagu Samba, Mysore Malli, Pal Kudai Vazhai, Ramakali, Semballai, Thooyamalli, Tulasi Vasanai Seeraga Samba, Vadakathi Samba, Vaigunda White, Vallan Samba, Vandana, Vasanai Seeraga Samba. |

**Supplementary Table. 4** MGIDI values and ranking of rice genotypes for multi trait selection under heat stress

| **Genotypes** | **MGIDI Value** | **Rank** | **Genotypes** | **MGIDI value** | **Rank** |
| --- | --- | --- | --- | --- | --- |
| TRY 1 | 4.98 | 1 | OTTUKICHADI | 8.09 | 41 |
| RNR 15048 | 5.62 | 2 | RAKTHASALI | 8.12 | 42 |
| TPS 5 | 5.76 | 3 | SAHBHAGI DHAN | 8.12 | 43 |
| INDHURANI | 6.20 | 4 | MANI SAMBA | 8.12 | 44 |
| ANNA R 4 | 6.55 | 5 | CHENGALPATTU SIRUMANI | 8.15 | 45 |
| SIVAPUKAVUNI | 6.76 | 6 | VASANAI SEERAGA SAMBA | 8.18 | 46 |
| TKM 15 | 6.95 | 7 | TULASI VASANAI SEERAGA SAMBA | 8.20 | 47 |
| TKM 13 | 6.95 | 8 | THOOYAMALLI | 8.20 | 48 |
| KNM 1638 | 6.98 | 9 | IR50 | 8.21 | 49 |
| BHAVANI | 6.98 | 10 | NAVARA | 8.25 | 50 |
| CO56 | 6.99 | 11 | MPR 606 | 8.27 | 51 |
| KALURUNDAIYAN | 7.07 | 12 | TKM 9 | 8.35 | 52 |
| ANNAI KOMBAN | 7.12 | 13 | ADT 46 | 8.41 | 53 |
| KALLUPUTHAN | 7.14 | 14 | RAMAKALI | 8.42 | 54 |
| NEI KICHADI | 7.18 | 15 | ASD 37 | 8.42 | 55 |
| ADUKKAN | 7.22 | 16 | MILAGU SAMBA | 8.48 | 56 |
| ADT 53 | 7.26 | 17 | KATTUYANAM | 8.54 | 57 |
| CHINNAR | 7.37 | 18 | CHINAADUKUNEL | 8.56 | 58 |
| MTU 1156 | 7.37 | 19 | POLINEL | 8.59 | 59 |
| CO51 | 7.38 | 20 | SEMBULI SAMBA | 8.62 | 60 |
| ASD 16 | 7.46 | 21 | POONGAR | 8.70 | 61 |
| KULIYADICHAN | 7.51 | 22 | ADT 37 | 8.84 | 62 |
| NAGINA 22 | 7.71 | 23 | 54R60 | 8.86 | 63 |
| IR 20 | 7.71 | 24 | KARUNKURUVAI | 8.91 | 64 |
| KAMBAN SAMBA | 7.71 | 25 | VALLAN SAMBA | 8.97 | 65 |
| VELLAI KUDAI VAZHAI | 7.73 | 26 | CO54 | 8.98 | 66 |
| KARUTHUKAR | 7.74 | 27 | VADAKATHI SAMBA | 8.98 | 67 |
| ADT 39 | 7.75 | 28 | KANDHASALA | 9.03 | 68 |
| WHITE PONNI | 7.77 | 29 | SATHAYU | 9.04 | 69 |
| NLR 3238 | 7.81 | 30 | MYSORE MALLI | 9.20 | 70 |
| TRY 5 | 7.81 | 31 | PADU 101 | 9.21 | 71 |
| NATTU BASMATI | 7.83 | 32 | KUTHIRAIVALLI SAMBA | 9.22 | 72 |
| VAIGUNDA RED | 7.83 | 33 | KADAIKALUTHAN | 9.26 | 73 |
| PR 128 | 7.89 | 34 | VAIGUNDA WHITE | 9.26 | 74 |
| KOCHIN SAMBA | 7.91 | 35 | SIVAN SAMBA | 9.43 | 75 |
| VARAKAL | 7.91 | 36 | BHUTHAKAIMA | 9.52 | 76 |
| PR 47 | 7.99 | 37 | CO55 | 9.56 | 77 |
| AKASHYA | 8.00 | 38 | VANDANA | 9.80 | 78 |
| PAL KUDAI VAZHAI | 8.03 | 39 | ILLUPAIPOO SAMBA | 9.92 | 79 |
| SEMBALLAI | 8.04 | 40 | ADT 54 | 10.10 | 80 |

**Supplementary Table. 5 Genotype specific flowering window dates, duration and thermal exposure across normal and heat stress environments (Excel)**

**Supplementary Table. 6 Flowering group classification of rice genotypes based on pooled normal environment days to 50% flowering (DFF)**

| **Genotypes** | **Normal DFF** | **Flowering group** | **Genotypes** | **Normal DFF** | **Flowering group** |
| --- | --- | --- | --- | --- | --- |
| 54R60 | 99 | Intermediate flowering | MTU 1156 | 100 | Intermediate flowering |
| ADT 37 | 108 | Late flowering | Mysore malli | 102 | Intermediate flowering |
| ADT 39 | 103 | Late flowering | Nagina 22 | 77 | Early flowering |
| ADT 46 | 109 | Late flowering | Nattu basmati | 95 | Early flowering |
| ADT 53 | 87 | Early flowering | Navara | 94 | Early flowering |
| ADT 54 | 104 | Late flowering | Nei kichadi | 93 | Early flowering |
| adukkan | 96 | Intermediate flowering | NLR 3238 | 96 | Intermediate flowering |
| Akashya | 92 | Early flowering | Ottukichadi | 96 | Early flowering |
| Anna R 4 | 96 | Early flowering | Padu 101 | 111 | Late flowering |
| annai komban | 88 | Early flowering | Pal kudai vazhai | 112 | Late flowering |
| ASD 16 | 79 | Early flowering | Polinel | 105 | Late flowering |
| ASD 37 | 109 | Late flowering | Poongar | 109 | Late flowering |
| Bhavani | 106 | Late flowering | PR 128 | 98 | Intermediate flowering |
| Bhuthakaima | 99 | Intermediate flowering | PR 47 | 100 | Intermediate flowering |
| Chengalpattu sirumani | 124 | Late flowering | Rakthasali | 96 | Intermediate flowering |
| Chinaadukunel | 103 | Late flowering | Ramakali | 94 | Early flowering |
| Chinnar | 103 | Late flowering | RNR 15048 | 102 | Intermediate flowering |
| CO51 | 94 | Early flowering | Sahbhagi Dhan | 96 | Early flowering |
| CO54 | 96 | Intermediate flowering | sathayu | 110 | Late flowering |
| CO55 | 101 | Intermediate flowering | semballai | 98 | Intermediate flowering |
| CO56 | 89 | Early flowering | sembuli samba | 107 | Late flowering |
| Illupaipoo samba | 111 | Late flowering | Sivan samba | 113 | Late flowering |
| Indhurani | 100 | Intermediate flowering | Sivapukavuni | 100 | Intermediate flowering |
| IR 20 | 103 | Late flowering | Thooyamalli | 93 | Early flowering |
| IR50 | 86 | Early flowering | TKM 13 | 100 | Intermediate flowering |
| Kadaikaluthan | 99 | Intermediate flowering | TKM 15 | 93 | Early flowering |
| Kalluputhan | 99 | Intermediate flowering | TKM 9 | 97 | Intermediate flowering |
| Kalurundaiyan | 82 | Early flowering | TPS 5 | 92 | Early flowering |
| kamban samba | 101 | Intermediate flowering | TRY 1 | 103 | Intermediate flowering |
| Kandhasala | 103 | Late flowering | TRY 5 | 98 | Intermediate flowering |
| Karunkuruvai | 100 | Intermediate flowering | Tulasi vasanai seeraga samba | 85 | Early flowering |
| Karuthukar | 98 | Intermediate flowering | Vadakathi samba | 86 | Early flowering |
| kattuyanam | 96 | Intermediate flowering | Vaigunda red | 96 | Early flowering |
| KNM 1638 | 105 | Late flowering | vaigunda white | 93 | Early flowering |
| Kochin samba | 92 | Early flowering | Vallan samba | 95 | Early flowering |
| Kuliyadichan | 108 | Late flowering | Vandana | 105 | Late flowering |
| kuthiraivalli samba | 117 | Late flowering | varakal | 95 | Early flowering |
| Mani samba | 89 | Early flowering | Vasanai seeraga samba | 95 | Early flowering |
| Milagu samba | 107 | Late flowering | Vellai kudai vazhai | 86 | Early flowering |
| MPR 606 | 108 | Late flowering | white ponni | 107 | Late flowering |

**Supplementary Table 7. Genotype wise mean biological yield, single plant yield (SPY) and harvest index (HI%) of rice genotypes and normal and stress condition**

| Genotypes | Normal biological yield | Normal SPY | Normal HI % | Stress Biological yield | Stress SPY | Stress HI% |
| --- | --- | --- | --- | --- | --- | --- |
| 54R60 | 136.55 | 32.26 | 20.40 | 62.09 | 20.10 | 33.20 |
| ADT 37 | 102.26 | 23.79 | 23.19 | 100.46 | 14.42 | 14.41 |
| ADT 39 | 229.01 | 19.44 | 10.53 | 110.59 | 11.38 | 10.30 |
| ADT 46 | 112.55 | 30.00 | 36.03 | 75.91 | 18.07 | 29.64 |
| ADT 53 | 214.54 | 52.79 | 38.26 | 81.20 | 13.59 | 14.93 |
| ADT 54 | 258.62 | 20.26 | 8.16 | 184.55 | 13.94 | 8.15 |
| ADUKKAN | 249.52 | 47.44 | 25.42 | 85.00 | 15.78 | 20.31 |
| AKASHYA | 215.70 | 31.33 | 18.32 | 81.52 | 15.50 | 23.72 |
| ANNA R 4 | 145.88 | 31.11 | 27.57 | 62.99 | 20.46 | 32.45 |
| ANNAI KOMBAN | 444.84 | 41.03 | 11.48 | 176.08 | 17.11 | 9.83 |
| ASD 16 | 100.29 | 16.21 | 23.86 | 46.27 | 12.66 | 27.53 |
| ASD 37 | 81.52 | 21.31 | 33.51 | 46.45 | 15.48 | 33.33 |
| BHAVANI | 185.35 | 27.74 | 21.60 | 66.78 | 17.62 | 26.29 |
| BHUTHAKAIMA | 623.44 | 50.83 | 8.19 | 223.32 | 13.30 | 5.57 |
| CHENGALPATTU SIRUMANI | 177.03 | 39.81 | 31.54 | 113.57 | 15.08 | 15.10 |
| CHINAADUKUNEL | 301.70 | 42.70 | 14.14 | 193.97 | 18.74 | 11.88 |
| CHINNAR | 127.03 | 35.93 | 33.82 | 71.54 | 13.27 | 18.53 |
| CO51 | 153.80 | 39.73 | 37.08 | 87.97 | 16.50 | 19.40 |
| CO54 | 94.31 | 36.19 | 40.11 | 61.51 | 17.93 | 33.53 |
| CO55 | 115.09 | 32.96 | 28.67 | 70.99 | 11.75 | 16.27 |
| CO56 | 90.01 | 24.60 | 27.14 | 95.46 | 13.14 | 13.90 |
| ILLUPAIPOO SAMBA | 299.72 | 26.56 | 9.85 | 160.22 | 5.62 | 3.69 |
| INDHURANI | 198.26 | 35.31 | 34.47 | 58.31 | 21.27 | 34.41 |
| IR 20 | 251.58 | 31.06 | 16.22 | 93.68 | 16.33 | 21.55 |
| IR 50 | 128.34 | 17.88 | 20.60 | 55.21 | 14.58 | 26.24 |
| KADAIKALUTHAN | 365.50 | 43.13 | 12.18 | 191.43 | 14.40 | 10.53 |
| KALLUPUTHAN | 187.91 | 44.14 | 31.32 | 93.36 | 18.99 | 20.59 |
| KALURUNDAIYAN | 235.38 | 38.71 | 18.60 | 114.50 | 14.80 | 14.17 |
| KAMBAN SAMBA | 165.67 | 28.57 | 22.78 | 59.79 | 15.23 | 32.18 |
| KANDHASALA | 199.44 | 7.15 | 3.66 | 145.80 | 5.44 | 3.91 |
| KARUNKURUVAI | 143.43 | 22.42 | 21.12 | 68.68 | 15.61 | 22.80 |
| KARUTHUKAR | 428.08 | 34.51 | 10.01 | 150.01 | 14.55 | 12.37 |
| KATTUYANAM | 275.19 | 28.01 | 10.65 | 172.84 | 12.14 | 6.81 |
| KNM 1638 | 193.83 | 34.56 | 26.47 | 67.72 | 16.46 | 25.81 |
| KOCHIN SAMBA | 350.51 | 29.11 | 8.34 | 269.12 | 16.60 | 6.47 |
| KULIYADICHAN | 245.80 | 25.15 | 13.26 | 129.59 | 16.62 | 13.08 |
| KUTHIRAIVALLI SAMBA | 230.95 | 30.88 | 16.08 | 128.25 | 12.80 | 10.02 |
| MANI SAMBA | 331.53 | 32.02 | 13.96 | 109.56 | 18.83 | 19.99 |
| MILAGU SAMBA | 608.28 | 24.31 | 5.78 | 127.87 | 9.88 | 7.88 |
| MPR 606 | 104.67 | 28.24 | 34.81 | 43.23 | 18.25 | 41.36 |
| MTU 1156 | 139.03 | 30.93 | 29.43 | 60.36 | 14.59 | 24.83 |
| MYSORE MALLI | 228.00 | 9.80 | 4.27 | 120.77 | 10.47 | 29.15 |
| NAGINA 22 | 124.38 | 29.83 | 27.46 | 90.13 | 16.65 | 18.65 |
| NATTU BASMATI | 155.60 | 23.11 | 20.54 | 62.93 | 19.97 | 32.11 |
| NAVARA | 330.82 | 23.30 | 7.43 | 228.53 | 22.41 | 15.07 |
| NEI KICHADI | 265.67 | 41.75 | 17.99 | 120.96 | 18.27 | 18.36 |
| NLR 3238 | 177.59 | 33.03 | 32.86 | 51.30 | 19.19 | 37.57 |
| OTTUKICHADI | 162.51 | 23.61 | 17.08 | 85.49 | 20.42 | 23.95 |
| PADU 101 | 166.09 | 25.87 | 23.50 | 62.53 | 13.36 | 28.78 |
| PAL KUDAI VAZHAI | 182.05 | 30.90 | 20.11 | 76.35 | 12.48 | 16.39 |
| POLINEL | 219.14 | 36.62 | 18.38 | 111.55 | 16.69 | 16.04 |
| POONGAR | 124.88 | 23.08 | 21.85 | 66.69 | 21.47 | 32.20 |
| PR 128 | 143.81 | 47.23 | 47.72 | 89.18 | 17.51 | 19.05 |
| PR 47 | 120.30 | 39.09 | 38.95 | 76.74 | 18.26 | 24.72 |
| RAKTHASALI | 164.98 | 22.62 | 16.25 | 76.75 | 16.72 | 20.91 |
| RAMAKALI | 297.00 | 26.44 | 9.67 | 161.66 | 14.61 | 9.17 |
| RNR 15048 | 176.43 | 33.06 | 23.36 | 93.66 | 15.18 | 16.16 |
| SAHBHAGI DHAN | 218.96 | 25.81 | 15.85 | 98.52 | 18.59 | 19.65 |
| SATHAYU | 74.83 | 24.96 | 42.29 | 62.63 | 12.74 | 21.98 |
| SEMBALLAI | 293.49 | 20.98 | 8.64 | 125.83 | 10.11 | 8.59 |
| SEMBULI SAMBA | 746.10 | 28.67 | 3.85 | 112.50 | 10.70 | 9.39 |
| SIVAN SAMBA | 159.19 | 26.24 | 20.94 | 73.95 | 11.95 | 16.12 |
| SIVAPUKAVUNI | 300.95 | 29.71 | 11.15 | 145.62 | 15.33 | 13.95 |
| THOOYAMALLI | 264.27 | 31.87 | 14.58 | 154.01 | 14.89 | 9.73 |
| TKM 13 | 271.82 | 47.65 | 22.96 | 98.14 | 17.60 | 21.20 |
| TKM 15 | 304.93 | 28.41 | 10.68 | 121.08 | 16.24 | 23.82 |
| TKM 9 | 145.44 | 42.13 | 36.28 | 63.39 | 21.64 | 39.41 |
| TPS 5 | 166.59 | 37.76 | 29.11 | 58.57 | 20.53 | 38.30 |
| TRY 1 | 320.97 | 40.22 | 16.23 | 136.39 | 20.60 | 16.98 |
| TRY 5 | 198.52 | 40.58 | 24.31 | 90.30 | 20.18 | 24.85 |
| TULASI VASANAI SEERAGA SAMBA | 191.59 | 30.55 | 19.26 | 120.51 | 5.62 | 4.80 |
| VADAKATHI SAMBA | 200.03 | 28.13 | 17.97 | 99.63 | 14.21 | 14.32 |
| VAIGUNDA RED | 219.37 | 41.66 | 23.96 | 91.20 | 16.13 | 17.77 |
| VAIGUNDA WHITE | 108.62 | 29.62 | 27.94 | 92.39 | 10.55 | 11.62 |
| VALLAN SAMBA | 287.57 | 39.23 | 13.44 | 189.72 | 15.60 | 9.28 |
| VANDANA | 92.83 | 25.32 | 29.01 | 71.62 | 10.76 | 14.93 |
| VARAKAL | 354.00 | 36.23 | 13.98 | 141.90 | 14.89 | 10.58 |
| VASANAI SEERAGA SAMBA | 292.11 | 24.92 | 8.79 | 162.49 | 9.42 | 5.94 |
| VELLAI KUDAI VAZHAI | 632.14 | 31.38 | 5.15 | 189.34 | 15.88 | 9.62 |
| WHITE PONNI | 226.39 | 30.74 | 14.54 | 147.32 | 18.08 | 13.25 |

**
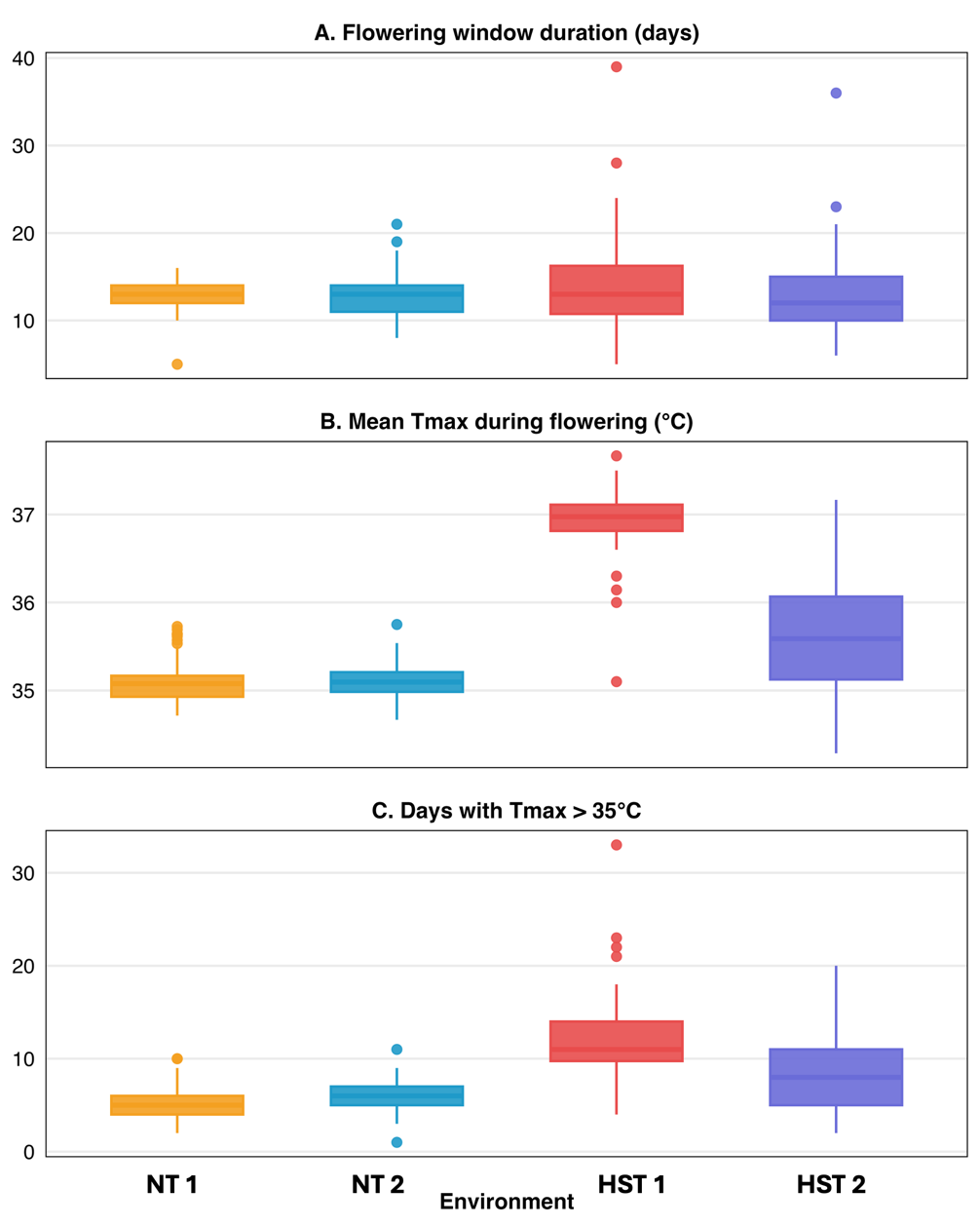
Supplementary Figure 1.** Boxplots showing flowering window duration and thermal exposure across normal and heat stress environments. A) Shows flowering window duration (days), B) shows mean maximum temperature (Tmax °C) during flowering window and C) shows the number of days with Tmax > 35°C. NT1 and NT2 represents normal environment, whereas HST 1, HST 2 represents the heat stress environments. The figure shows that the stress environments, particularly HST 1 exposed genotypes to greater reproductive stage thermal load that the normal environments.
